# The Impact of Methane on Microbial Communities at Marine Arctic Gas Hydrate Bearing Sediment

**DOI:** 10.3389/fmicb.2020.01932

**Published:** 2020-09-24

**Authors:** Vincent Carrier, Mette M. Svenning, Friederike Gründger, Helge Niemann, Pierre-Antoine Dessandier, Giuliana Panieri, Dimitri Kalenitchenko

**Affiliations:** ^1^Department of Arctic and Marine Biology, The Arctic University of Norway, Tromsø, Norway; ^2^Centre for Arctic Gas Hydrate, Environment and Climate, The Arctic University of Norway, Tromsø, Norway; ^3^Department of Bioscience, Arctic Research Centre, Aarhus University, Aarhus, Denmark; ^4^Department of Marine Microbiology and Biogeochemistry, Royal Netherlands Institute for Sea Research, and Utrecht University, Den Burg, Netherlands; ^5^Department of Earth Sciences, Faculty of Geosciences, Utrecht University, Utrecht, Netherlands

**Keywords:** Arctic, methane seeps, prokaryotes, methanotrophs, ANME, Sulfate-reducing bacteria, eukaryotes, foraminifera

## Abstract

Cold seeps are characterized by high biomass, which is supported by the microbial oxidation of the available methane by capable microorganisms. The carbon is subsequently transferred to higher trophic levels. South of Svalbard, five geological mounds shaped by the formation of methane gas hydrates, have been recently located. Methane gas seeping activity has been observed on four of them, and flares were primarily concentrated at their summits. At three of these mounds, and along a distance gradient from their summit to their outskirt, we investigated the eukaryotic and prokaryotic biodiversity linked to 16S and 18S rDNA. Here we show that local methane seepage and other environmental conditions did affect the microbial community structure and composition. We could not demonstrate a community gradient from the summit to the edge of the mounds. Instead, a similar community structure in any methane-rich sediments could be retrieved at any location on these mounds. The oxidation of methane was largely driven by anaerobic methanotrophic Archaea-1 (ANME-1) and the communities also hosted high relative abundances of sulfate reducing bacterial groups although none demonstrated a clear co-occurrence with the predominance of ANME-1. Additional common taxa were observed and their abundances were likely benefiting from the end products of methane oxidation. Among these were sulfide-oxidizing Campilobacterota, organic matter degraders, such as Bathyarchaeota, Woesearchaeota, or thermoplasmatales marine benthic group D, and heterotrophic ciliates and Cercozoa.

## Introduction

Cold seep microbial communities thrive where geofluids, characterized by high concentrations of hydrocarbons, in particular methane (CH_4_), provide a primary energy source for these organisms (Boetius et al., 2000; Orphan et al., 2002; Niemann et al., 2013). These geofluids and/or free gas migrate upward through faults, cracks, and sediment pores that provide a transport vector from sub-seafloor reservoirs to the seafloor. The origin of methane can be either from the geological cracking of organic matter at high temperature or from biologically mediated decomposition of organic matter (Schoell, 1988; Joye et al., 2010). Under certain thermobaric conditions, CH_4_ forms gas hydrates, i.e., an ice-like lattice comprising molecules of CH_4_ trapped in crystalline cages of water molecules. The formation or the dissociation of gas hydrates can modify the seafloor morphology, and subsequently can lead to the genesis of pockmarks, craters, and gas domes (Vogt et al., 1994; Hovland and Svensen, 2006; Koch et al., 2015; Portnov et al., 2016; Serov et al., 2017; Waage et al., 2019).

The CH_4_ present in the fluid can be oxidized aerobically or anaerobically (Krüger et al., 2005; James et al., 2016). In aerobic environments, the oxidation of CH_4_ is driven by methane oxidizing bacteria that utilize oxygen as an electron acceptor. Most of them are associated with the alpha and gamma proteobacteria, but also with Verrucomicrobia or *Crenothrix* (Hanson and Hanson, 1996; Knief, 2015). Nevertheless, microbial activity at the cold seep seafloor rapidly depletes the available oxygen in marine sediments and limits its penetration depth to a small surface layer, usually of a few millimeters thickness at most (Niemann et al., 2006, 2009; Reeburgh, 2007; Boetius and Wenzhöfer, 2013). In the absence of oxygen, methane is oxidized anaerobically through a process that has been termed the anaerobic oxidation of methane (AOM; Reeburgh, 2007). Anaerobic oxidation of methane is driven by anaerobic methanotrophic Archaea (ANME) and so far, three main ANME clades of phylogenetically distinct groups were detected: ANME-2 and ANME-3 are placed within the methanosarcinales, while ANME-1 forms a distinct group within the Halobacterota (Knittel and Boetius, 2009; Quast et al., 2012; Yilmaz et al., 2014). The phylogenetic dissimilarity of these ANME groups suggests different levels of tolerance to various environmental parameters. Previous study results suggested that ANME-2 might be more sensitive than ANME-1 to high concentrations of sulfide and low concentrations of sulfate (Timmers et al., 2015; Bhattarai et al., 2018). The ANME-2 group would then often be limited to the layers at the sulfate-methane transition zone (SMTZ) and ANME-1 would dominate in more sulfidic sediments, at deeper layers (Knittel et al., 2005; Roalkvam et al., 2012). Nevertheless, ANME-2 groups were also retrieved in sulfide-rich sediments (for example at the Hydrate Ridge; Knittel et al., 2003), insinuating the impact of other factors on the observed stratification of ANME groups. Additional environmental conditions that were suggested to select for differential ANME groups include temperature (Nauhaus et al., 2005; Rossel et al., 2011), salinity (Maignien et al., 2013), or CH_4_ flux rates (Girguis et al., 2005; Yanagawa et al., 2011; Marlow et al., 2014).

Most ANME use sulfate, but some were also found to use iron, manganese, and nitrite/nitrate as electron acceptors (Beal et al., 2009; Ettwig et al., 2010, 2016; Hu et al., 2014). Reduction of sulfate at the SMTZ generally requires sulfate reducing bacteria (SRB) and a syntrophic consortium with ANME that are commonly found as AOM drivers (Boetius et al., 2000; Wegener et al., 2015). However, in the last decade, community studies of methanotrophs have shown evidence of free-living ANME cells particularly assigned to the ANME-1 group, but also to the ANME-2 group, that might perform sulfate reduction alone (Orphan et al., 2002; Knittel et al., 2005; Roalkvam et al., 2011; Milucka et al., 2012; Stokke et al., 2012; Gründger et al., 2019).

The AOM coupled with sulfate reduction generates HS^–^ which can subsequently be oxidized by sulfide-oxidizing bacteria, such as the bacterial mat forming *Beggiatoa* or Campilobacterota species. Some chemoautotrophs can also be present as intracellular and extracellular symbionts within larger fauna, but also in the eukaryotic euglenozoans and ciliates (Buck et al., 2000; Rinke et al., 2006). Additionally, a higher bacterial and archaeal biomass becomes a trophic basis for grazing megafauna or microbial eukaryotes, including diverse bacterivore ciliates, Cercozoa, and stramenopiles (Werne et al., 2002; Takishita et al., 2007, 2010; Niemann et al., 2013). Potentially parasitic or pathogenic eukaryotes, such as Apicomplexa, Ichthyosporea, and fungi, are also likely to benefit from the denser faunal community (Atkins et al., 2002; Takishita et al., 2006).

In the Arctic, gas hydrate bearing domes were observed 50 km south of Svalbard in Storfjordrenna, at ∼390 m below sea level (Serov et al., 2017). They are referred to as pingos, after similar terrestrial features observed in glacial valleys (Mackay, 1998), although they differ by their formation (i.e., gas hydrates instead of regular water ice; Serov et al., 2017). At the water depth of the gas hydrate pingos (GHP; ∼390 m, ∼0.5–2.5°C bottom water T°C), the gas hydrates remain within the gas hydrate stability zone (GHSZ), but are close to its upper limit and are sensitive to even small changes of temperature and pressure (Hong et al., 2018). Hydroacoustic observations have revealed acoustic flares originating from methane gas bubbles in the water column. These were primarily located at the summit on four of the five Storfjordrenna pingos. The dating of methane derived authigenic carbonates suggested that CH_4_ seepage has been active for several thousand years (Serov et al., 2017). Visual observations have revealed a higher biomass in sediments of the pingos compared to the surrounding seafloor (Åström et al., 2018). This can be explained by the presence of a carbonate crust induced by AOM, which offers a hard substrate for the attachment of benthic organisms, such as sponges and anemones (Niemann et al., 2005; Cordes et al., 2010; Vaughn Barrie et al., 2011).

Past investigations at the Storfjordrenna pingos have primarily addressed the geochemical conditions (Serov et al., 2017; Hong et al., 2018) or the biodiversity of larger fauna (Sen et al., 2018; Åström et al., 2018), but the microbial community structure remains mostly unknown [with the exception of a biofilm retrieved within deeper sediments at the pingos (Gründger et al., 2019)]. At a circular seep further south, the Haakon Mosby Mud Volcano (HMMV), the composition of the bacterial and archaeal communities varied between concentric zones around the apex of the edifice, i.e., along a methane flux/concentration gradient (Niemann et al., 2006; Lösekann et al., 2007). In Storfjordrenna, the gas flares at the summit of the structures could suggest a similar concentric arrangement of microbial habitats. However, these pingos contrast with HMMV by presenting a multitude of small geological fractures and gas hydrates chaotically distributed around the structures through which methane migrates to the seafloor surface (Hong et al., 2018; Waage et al., 2019).

Our study aimed at determining spatial variations in the microbial community structure along a gradient from the apex to the edge of three pingos. We addressed key environmental factors that are influencing the prokaryotic and eukaryotic community structures and their spatial distribution. Finally, we identified key taxa characteristics for these Arctic CH_4_-rich environments, demonstrating the uniqueness of this ecosystem.

## Materials and Methods

### Study Site

The sampling site was located in the Arctic Ocean at the mouth of Storfjordrenna, 50 km south of Svalbard at approximatively 390 m water depth (Serov et al., 2017). A group of 5 GHPs (∼10 m high, 500 m in width) distributed on the seabed over a 2.5 km^2^ area were recently found^1^. Hydroacoustic surveys and real time visually guided observations with a TowCam-Multicore (see footnote 1) System (TC-MC) and Remotely Operated Vehicle (ROV) dives^2^ have revealed acoustic flares of gas bubbles consisting predominantly of CH_4_ and being emitted from 4 of the 5 GHPs. One structure (GHP 5) did not show any visible flares or gas hydrate in sediments (Serov et al., 2017). During the sampling campaigns for this study, seep activity at the different sampling sites was assessed by tracking flares through hydro acoustic surveys with a multibeam echosounder (Kongsberg Simrad EM 302) or by visual observations using a TC-MC system configuration (Panieri et al., 2017).

### Sampling Procedure

**FIGURE 1:**
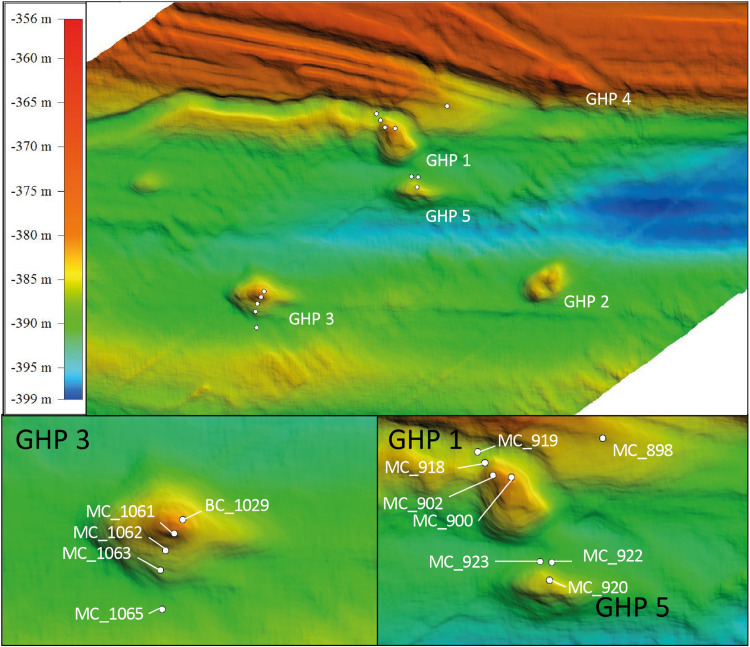
Geological dome structures referred to as Gas Hydrate Pingo (GHP) located at the mouth of Storfjordrenna, 50 km south of Svalbard. The upper panel gives an overview of this area. The lower panels show the three selected GHPs for this study. White dots represent locations of the different sediment cores at GHPs 1, 3, and 5 using a multicore system. Core MC_898 was sampled as reference site and core BC_1029. GHP 3 was taken using a blade core mounted on a ROV to sample in the vicinity of a methane gas flare.

Field campaigns were conducted with RV Helmer Hanssen and sediment cores at the GHP 3 and at GHPs 1 and 5 were taken in June 2016(see footnote 2) and June 2017^3^, respectively. Cores were taken along a spatial gradient from the apex of the geological feature to its edge. Core IDs MC_900 (apex), MC_902, MC_918, and MC_919 (edge) were taken at GHP 1. Core IDs MC_1061 (apex), MC_1062, MC_1063, and MC_1065 (edge) were collected at GHP 3 while core IDs MC_920 (apex), MC_922, and MC_923 (edge) were collected at GHP 5 (Figure 1). A reference core (core ID 898) was retrieved at one kilometer away from the closest GHP. The use of the multicore system KC Denmark DK8000 integrated with a TC-MC with a real time transmission of images (Daniel et al., 2003) allowed for the collection of six 60 cm long real time visually guided cores. The combined TC-MC was used to visually survey and sample sediments from the study site and the sediment recovery varied between 15 and 40 cm. Exceptionally, core ID BC_1029 was taken using a blade core mounted on a Sperre Subfighter 30k ROV to target directly sediments in close vicinity to a CH_4_ gas flare at GHP 3 in June 2016.

### Porewater Geochemistry

Porewater geochemistry was measured for all cores, and data for BC_1029 and MC_1063 were collected from Hong et al. (2020). CH_4_ concentrations were measured with a head space technique and gas chromatography (Thermoscientific Trace 1310) equipped with a flame ionization detector (Hoehler et al., 2000; Panieri et al., 2017). For this, we extruded 3 mL of bulk sediments per 2 cm intervals in all cores which were immediately transferred to a 20 mL headspace vial with 7 mL of NaOH solution (1 M) and two glass beads, and instantly capped. Samples were analyzed subsequently to an equilibration period of 24 h and are represented as concentration per sediment volume. Sediment porosity was determined from weight and volume measurements as presented in Boyce et al. (1973).

Dissolved iron (Fe^2+^), alkalinity, total sulfide (ΣHS), sulfate (SO_4_^2–^), and dissolved inorganic carbon (DIC) were measured from a neighboring core of the multicore system, recovered during the same sampling round. Using rhizon samplers (Seeberg-Elverfeldt et al., 2005), up to 18 mL of porewater was collected at each cm in the upper 10 cm and at intervals of 4–10 cm in the lower part of the core. Alkalinity and Fe^2+^ were measured onboard by titration and by spectrophotometry, respectively (Hong et al., 2017). SO_4_^2–^ was measured onshore using ion chromatography (Hong et al., 2017), while ΣHS was measured using a spectrophotometer at a wavelength of 670 nm (Cline, 1969). A detailed protocol on the measurement of ΣHS can be found in the Supplementary Material of Hong et al. (2020). Due to equipment availability on the two field cruises, ΣHS and DIC concentrations were not measured for all sediment cores while alkalinity and Fe^2+^ concentrations were only measured for a selection of sediment layers (Supplementary Tables 1–5).

### DNA Extraction, Sequencing, and Sequences Analyses

Sediment cores were extruded and 2 cm thick layers were transferred in Whirl-Pak^®^ sterile sampling bags (Nasco, United States) and stored at -80°C. Following the measurements of the different environmental parameters in the laboratory, 55 of these samples were selected for amplicon libraries sequencing. These samples were selected at regular depths (surface, ∼5, ∼10, and ∼15 cm) and at clear geochemical interfaces as detected by porewater geochemical gradients (e.g., SMTZ). In a cold room (4°C), sediments were manually ground in liquid nitrogen using a sterilized mortar. The DNA was extracted using the DNeasy PowerSoil Kit (Qiagen, Germany). The manufacturer protocol was followed, except that the samples were placed in G2 DNA/RNA Enhancer beads tubes (Ampliqon, Denmark) for physical lysis (Jacobsen et al., 2018) instead of the kit lysis tubes. Once the DNA samples were quality checked using electrophoresis gels, the DNA concentrations were measured using a NanoDrop^TM^ 2000 spectrophotometer (Thermo Fisher Scientific, United States) and normalized before being sent to the IMGM Laboratories GmbH for library preparation and amplicon sequencing. For each sample, eukarya were amplified using 18S rDNA degenerate primers to target the V4 region, and bacteria and archaea were amplified using 16S rDNA degenerate primers to target the V3-V4 region (Supplementary Table 6). Library generation was conducted in accordance with the company’s protocols before being sequenced using a Miseq System (Illumina inc., United States). Paired-end nucleotide reads were deposited at Sequence Read Archive Genebank^4^ as BioProject accession number PRJNA593930.

Paired-end reads were meticulously processed and the workflow was derived from the USEARCH suggested protocol^5^. Pairs were merged before being length trimmed and quality filtered with USEARCH v10.0.240. Thereafter, operational taxonomic units (OTUs) were constructed using the UPARSE-OTU greedy algorithm at 97% pairwise sequence identity. Singleton OTUs were removed and taxonomy was assigned using the method Wang implemented in Mothur to the SILVA database release 138 (Edgar, 2010; Quast et al., 2012; Yilmaz et al., 2014). Sequences that were not classified to their domain were discarded prior to further statistical analyses. Finally, sequences from multicellular organisms are likely detected within the 18S rDNA libraries and therefore OTUs that were assigned to Metazoan groups and unclassified eukaryotes were discarded to focus only on the microbial community.

### Statistical Analyses

Archaeal, bacterial, and eukaryotic libraries were rarefied at 8900, 4700, and 1300 sequences, respectively, corresponding to the lowest number of sequences in one sample. Preliminary analyses of the libraries demonstrated a large fraction of OTUs that contained just a few sequences in a sample, especially for the bacterial communities (Figure 6). In this study, we aimed to determine the distribution patterns of key microbes. The inclusion of a large fraction of rarer taxa in the diversity analyses, despite sharp gradients in the dominating OTUs, prevented the visualization of these gradients of community changes. Therefore, only OTUs containing at least 1% of the overall sequences of one sample were kept for further statistical analyses.

For the bacterial and archaeal communities, beta diversity, measuring changes in the composition of communities between different samples, was calculated on the relative abundance of the selected abundant OTUs using the Bray–Curtis dissimilarity index implemented in the Vegan v2.5-5 package on R (Oksanen et al., 2019). Clusters of sediment samples sharing similar OTUs abundance and composition for both domains of life were formed at a dissimilarity index of ca. 0.5–0.6. For each cluster, the relative abundance of each OTU was averaged and used to build a doughnut diagram with the R package ggplot2 v3.2.1. Thereafter, distance-based redundancy analyses (dbRDA) were performed to reveal whether the environmental parameters measured had an impact on the observed community dissimilarity between the different sediment cores. A dissimilarity matrix was built using the Bray–Curtis dissimilarity index. As the environmental parameters differed between the GHPs, and the fact that missing values can affect the outcome of the analyses, the dbRDA were performed and presented for each GHP separately. Environmental parameters were logarithmically transformed and standardized through Z scoring (Legendre and Legendre, 1998). The significance of the resulting axis from the dbRDA was evaluated through permutation tests (*n* = 999). Both functions for dbRDA and permutations tests are implemented in the Vegan v2.5-5 package on R (Oksanen et al., 2019).

For the eukaryotic libraries, biodiversity analyses were likely affected by the removal of sequences assigned to Metazoa, as in some samples they could represent on average 40% of the sequences. Furthermore, a large fraction of the community structure at the GHPs site was dominated by reads assigned to photosynthetic eukaryotes that might have originated from the sedimentation of phytoplankton cells, undermining any subsequent attempts at describing the structure of the eukaryotic communities thriving at the GHPs and evaluating the impact of environmental factors on the biodiversity (Rey and Rune Skjoldal, 1987). Therefore, a different approach was used for the eukaryotic libraries and we emphasized instead on the contrast of the abundant OTUs composition between the reference site and CH_4_-rich sediments. To do so, once sequences assigned to Metazoa or unclassified eukaryotes were removed and eukaryotic libraries were rarefied, OTUs that were abundant at the reference site were subtracted and presented separately. We hypothesized that the remaining abundant OTUs would be indicators of taxonomic groups influenced by local conditions at the GHPs. Analyses on the relative abundances of these taxonomic groups were calculated using the Bray-Curtis dissimilarity index (Oksanen et al., 2019) and clusters of sediment samples were formed at a dissimilarity index of 0.5–0.6.

### Benthic Foraminiferal Analyses

We observed that the relative abundances of certain prokaryotic taxonomic groups, including the genus *Sulfurimonas*, increased in CH_4_-rich sediments. To ensure that the changes in relative abundances of these taxonomic groups were caused by the presence of CH_4_, we compared results from DNA sequences with an independent proxy for surface CH_4_-rich sediments. Agglutinated foraminifera are often observed in Arctic seas (Wollenburg and Mackensen, 1998; Jernas et al., 2018) and are particularly sensitive to cold seeps where they are very rare or even absent (Panieri and Sen Gupta, 2008; Martin et al., 2010; Dessandier et al., 2019). Accordingly, changes in their abundances can be used to assess the impact of CH_4_ seepage disturbance on the local biological communities. Foraminiferal samples (0–1 cm sediment depth) from GHP 1 were stored for 14 days at 4°C in a 2 g L^–1^ Rose Bengal solution in ethanol 96%, in order to identify the living (Schönfeld et al., 2012), or recently alive individuals (Rose Bengal stained foraminifera; Corliss, 1991). All samples were wet sieved using 63 and 125 μm mesh sieves and dried at 40°C (48 h). We considered “living” individuals as the ones characterized by a pink stain of all chambers in their test, with the exception of the last one. In case of doubt, the test was broken to investigate the staining of the endoplasm (Schönfeld et al., 2012). All benthic foraminiferal specimens from >125 μm size fraction were handpicked, identified, and counted. The density was calculated by dividing the number of agglutinated foraminiferal individuals (Supplementary Table 10) in each core by the surface of the core (5.02 × 10^3^ m^2^). The relationship between the density of agglutinated foraminiferal cells and the logarithm of the number of resampled *Sulfurimonas* sequences was tested using a linear model.

## Results

### Environmental Characterization and Geochemistry

**FIGURE 2:**
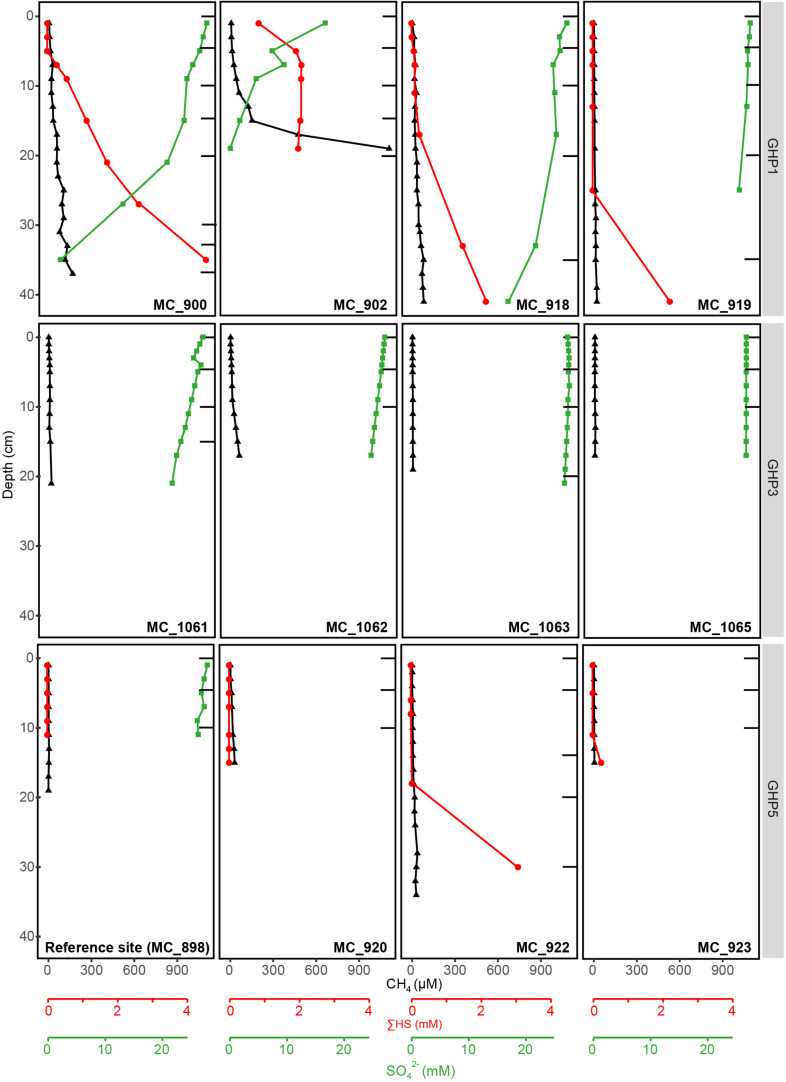
Geochemical profiles of the different sediment cores collected at the reference site (MC_898) and GHPs 1, 3, and 5, with the exception of BC_1029 that is presented in Figure 3. Profiles for CH_4_, SO_4_^2–^, and ΣHS at these sediment depths are given. Black bars correspond to the sediment layers from which the DNA was extracted and sequenced.

At the reference site, CH_4_ was nearly absent, gas flares were not detected on the echosounder, and CH_4_ sediment concentrations did not exceed 4 μM (Figure 2). ΣHS remained undetectable throughout the reference core, while measured concentrations of SO_4_^2–^ slightly decreased from 28 mM at the sediment surface to 26 mM at 11 cm below seafloor (bsf), correlating to seawater concentration of the Barents Sea. The seafloor was muddy and authigenic carbonates were not observed (Supplementary Table 4).

At GHP 1, gas flares and high CH_4_ sediment concentrations were suggestive of high CH_4_ seepage activity (Figures 1, 2). Dense patches of chemosynthetic organisms, such as siboglinids, as well as carbonate crusts colonized by anemones and sponges, were scattered across GHP 1 (Supplementary Table 4). Concentrations of CH_4_ were low in the sediment surface layer, ranging from 0.61 to 6.73 μM, and increased with depth in cores taken at the GHP 1 apex, reaching a maximum of 169 μM at 37 cmbsf in core MC_900 and 1109 μM at 19 cmbsf in core MC_902 (Figure 2). MC_902 was also characterized by a stronger depletion of SO_4_^2–^ with depth than at the reference site as concentration dropped below 5 mM at 15 cmbsf. With the decrease in SO_4_^2–^, ΣHS concentrations increased, peaking at 4558 and 2078 μM in MC_900 and MC_902, respectively. MC_918 was collected close to the rim of the GHP, where concentrations of CH_4_ and ΣHS increased with depth, but at lower concentrations than at cores taken near the apex of GHP 1. The SO_4_^2–^ concentrations values at MC_918 ranged from 27.8 mM at the surface to 25.9 mM at 19 cmbsf. MC_919 was taken outside the GHP, but close to its edge. Here, environmental parameters became more similar to the reference site. Low concentrations of CH_4_ (yet still slightly higher than at the reference site) were detectable and SO_4_^2–^ concentrations were only slightly lower than at the reference site and remained above 26 mM within this core.

**FIGURE 3:**
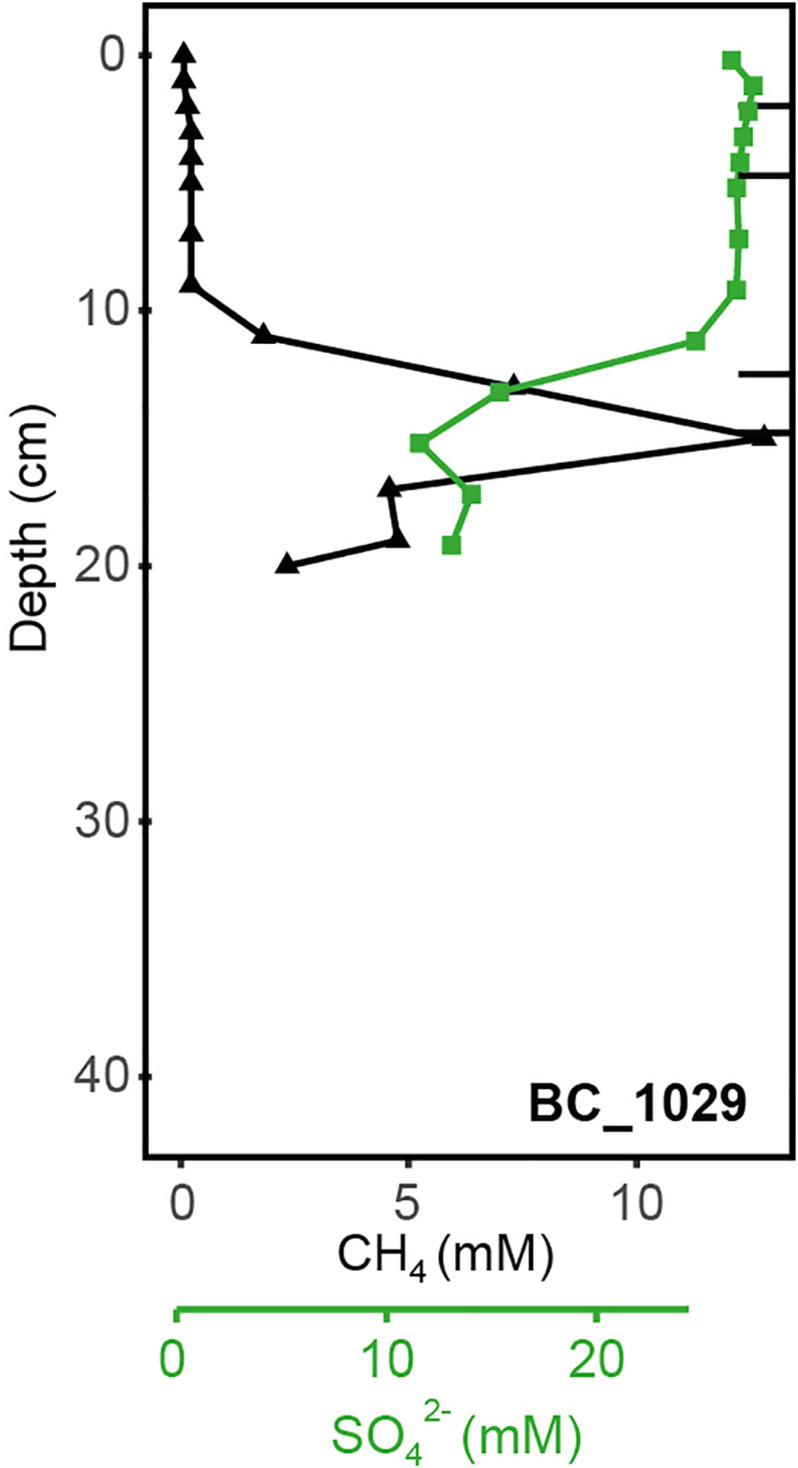
Geochemical profiles of the sediment core BC_1029 collected at GHP 3 near a CH_4_ gas flare. Profiles for CH_4_ and SO_4_^2–^ at these sediment depths are shown. Black bars correspond to the sediment layers from which the DNA was extracted and sequenced.

At GHP 3, BC_1029 had the highest CH_4_ concentrations of all sites, reaching up to 12.8 mM at 12 cmbsf (Figure 3). This core was taken in the vicinity of a CH_4_ gas flare (Figure 1). The four other cores from GHP 3 had lower CH_4_ concentrations than BC_1029 (<15 μM). Still, the cores MC_1061 and MC_1062, located close to the GHP 3 apex, had higher CH_4_ concentrations than cores MC_1063 and MC_1065, collected near the edge and outside GHP 3, respectively. SO_4_^2–^ maximum concentrations in the surface sediment layers were in the range of 27-28 mM for all cores, but the SO_4_^2–^ level decreased to 12.21 mM at 12 cmbsf and at 23.83 mM at 14 cmbsf for BC_1029 and MC_1061, respectively. Within other cores taken at GHP 3, the decreasing concentrations of SO_4_^2–^ showed a similar pattern to the reference site. Fe^2+^ concentrations were only measured in two cores (BC_1029 and MC_1063) and showed a sharp decrease at the sediment surface in core BC_1029, but remained high in core MC_1063, where it was depleted only at 20 cmbsf (Supplementary Table 2).

At GHP 5, similar CH_4_ concentrations in the upper sediment layer were measured in core MC_920 and at the apex of GHP 3 (Figure 2). However, gas flares were not visible on the echosounder at the apex of GHP 5. In addition, a CH_4_ concentration of ∼18 μM was measured at 19 cmbsf in core MC_922, occurring concomitantly with an increasing concentration of ΣHS. The seafloor was covered with hard surfaces, mostly ice raft debris, and colonized by anemones and sponges (Supplementary Table 5). Complementary information on visual observations at the sampling sites and on concentrations of Fe^2+^, alkalinity, and DIC are available as Supplementary Information (Supplementary Tables 4, 5).

### Taxonomy and Abundant OTUs

Once pair-ends reads were quality filtered, 8 129, 36 301, and 8184 OTUs were successfully assigned to the archaeal, bacterial, and eukaryotic domains, respectively (Supplementary Table 7). After rarefaction, within the archaeal OTUs, 87 were found to be abundant in at least one of the sediment layers collected from the reference site or the GHPs. Among the bacteria and eukaryotes, 107 and 140 abundant OTUs were identified, respectively.

### Composition Similarities of the Microbial Communities

**FIGURE 4:**
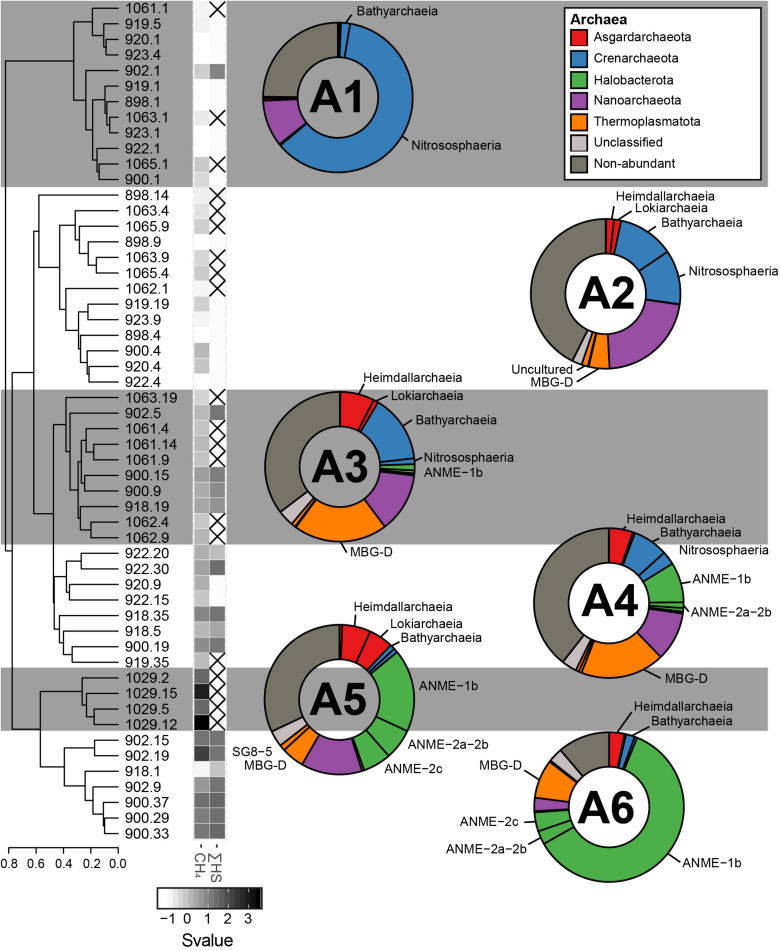
The sediment archaeal communities clustered in six different groups, calculated based on the Bray–Curtis dissimilatory index from the composition of abundant archaeal OTUs (A1, A2, A3, A4, A5, and A6). The averaged taxonomic composition of each cluster is illustrated in a doughnut chart with colors indicating taxa listed in the box. “Non-abundant” includes sequences assigned to archaeal OTUs that were not retrieved in abundance in this study. Finally, the heatmap gives standardized values (Svalue) of depth and of the logarithmic concentrations of CH_4_ and HS. Non-available data are represented by striped squares.

**FIGURE 5:**
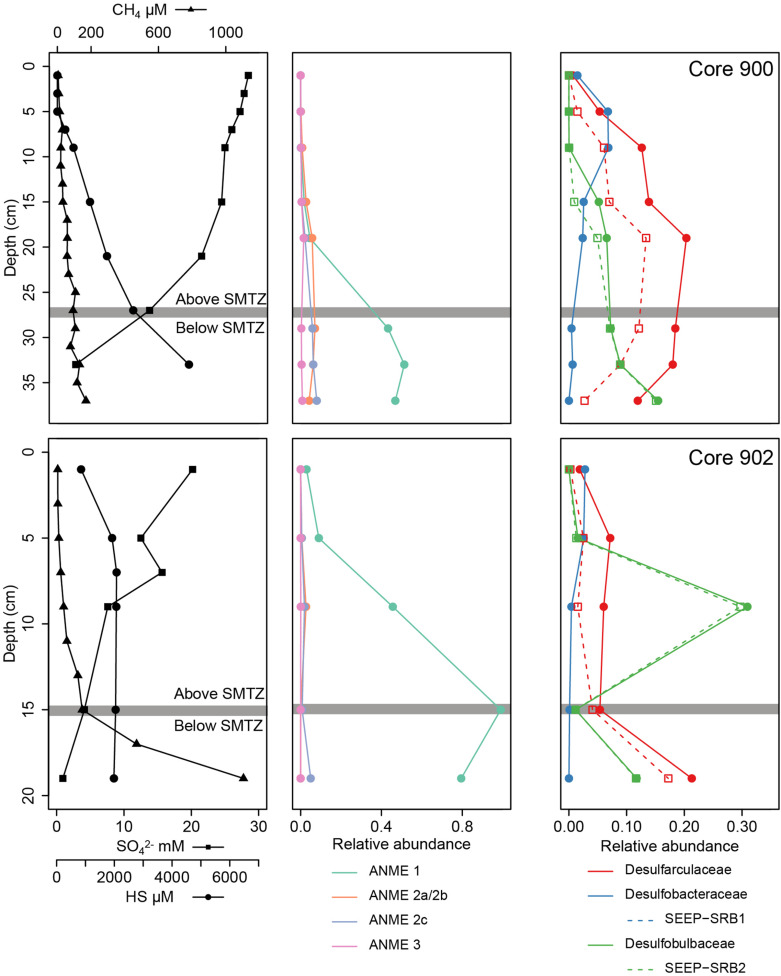
Physico-chemical and biological vertical profiles of the cores MC_900 and MC_902 (GHP 1) where a SMTZ was identified. For each core, the left figure presents the CH_4_, SO_4_^2–^, and HS concentration profiles. The centered and right figures present the relative abundances of archaeal methane oxidizers (ANME groups) and sulfate-reducing bacteria groups, respectively.

Based on the beta-diversity dissimilarity analyses in the GHP sediments (Figure 4), six different community clusters designated A1, A2, A3, A4, A5, and A6 were identified for the archaeal domain. Cluster A1 included nearly all surface sediment samples and was dominated by the Crenarchaeota Nitrososphaera and the Nanoarchaeota Woeserchaeales, with 61.4 and 10.0% of the total archaeal community, respectively. Sediment layers associated to the cluster A2 were from different depths, although most were collected between 4 and 10 cmbsf. The cluster A2 was characterized by a stronger dominance of Woeserchaeales (21.86%), Bathyarchaeia (12.14%), Nitrososphaera (11.73%), the marine benthic group D (MBG-D) within the Thermoplasmatota (4.49%), and Asgardarchaeota (3.35%). In addition, 2.1% of the sequences were associated to an unclassified archaeal OTU. The community of the cluster A3 was driven by the MBG-D (20.1%), the Bathyarchaiea (14.5%), and the Woesearchaeales (12.7%). The Asgardaeota groups of Heimdallarchaeia (7.3%) and Lokiarchaeia (1.1%), and the Halobacterota ANME-1 group (1.3%) were also predominant. The cluster A4 had a similar community composition to the cluster A3 and was dominated by the MBG-D (18.5%), the Wosearchaeales (10.8%), and ANME-1 (8.8%). Bathyarchaeia (7.7%) and Asgardarchaeota (5.8%) were also major components of the A4. The clusters A5 and A6 differed from the other groups particularly by a higher relative abundance of sequences associated to ANME groups. The cluster A5, representing sediment layers at the gas flare (core BC_1029) was mainly composed of ANME-1 (17.9 %), ANME-2a/-2b (6.6 %), and ANME-2c (6.1%). Other abundant taxonomic groups included Wosearchaeales (13.2 %) and the MBG-D (4.9%), in addition to the Asgardarchaeota Heimdallarchaeia (6.1%) and Lokiarchaeia (5.3%). The ANME communities of A6 was in contrast to A5 by a stronger dominance of ANME-1 (60.7%; Figure 7), in comparison to the ANME-2a-2b (2.9%) and ANME-2c (4.1%; Figure 5), were also abundant in the cluster A6 representatives from the MBG-D (8.3%).

**FIGURE 6:**
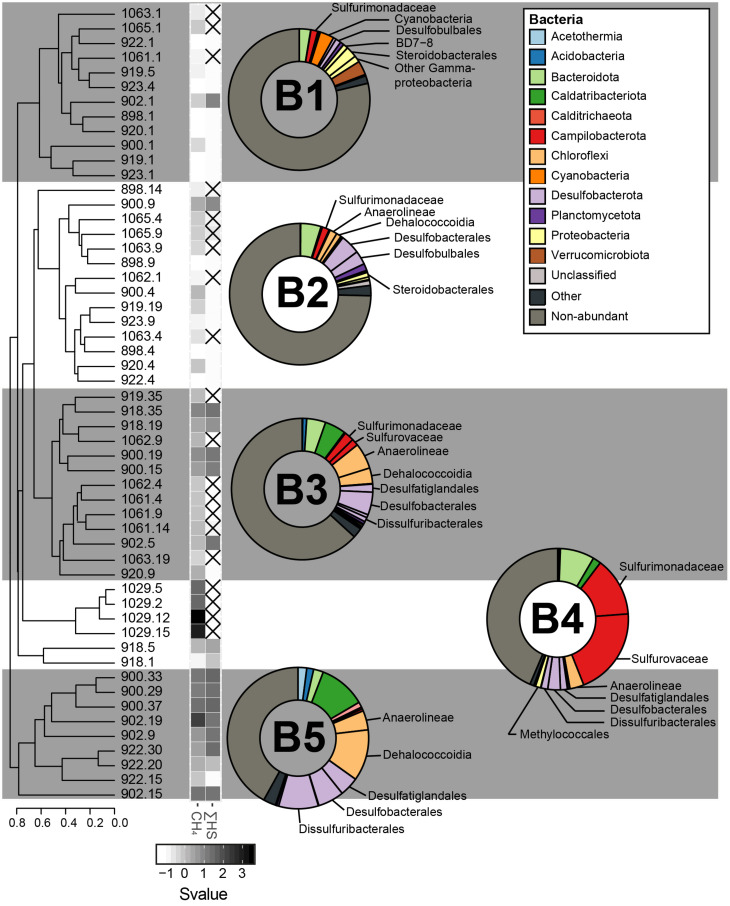
The sediment bacterial communities clustered in five different groups, calculated based on the Bray–Curtis dissimilatory index from the composition of abundant bacterial OTUs (B1, B2, B3, B4, and B5). The averaged taxonomic composition of each cluster is illustrated in a doughnut chart with colors indicating taxa listed in the box. “Other” relates to sequences that are assigned to OTUs abundant throughout the whole communities, but not within the illustrated cluster. As for the group “non-abundant”, it includes sequences assigned to bacterial OTUs that were not retrieved in abundance in this study. Finally, the heatmap gives standardized values (Svalue) of depth and of the logarithmic concentrations of CH_4_ and HS. Non-available data are represented by striped squares.

**FIGURE 7:**
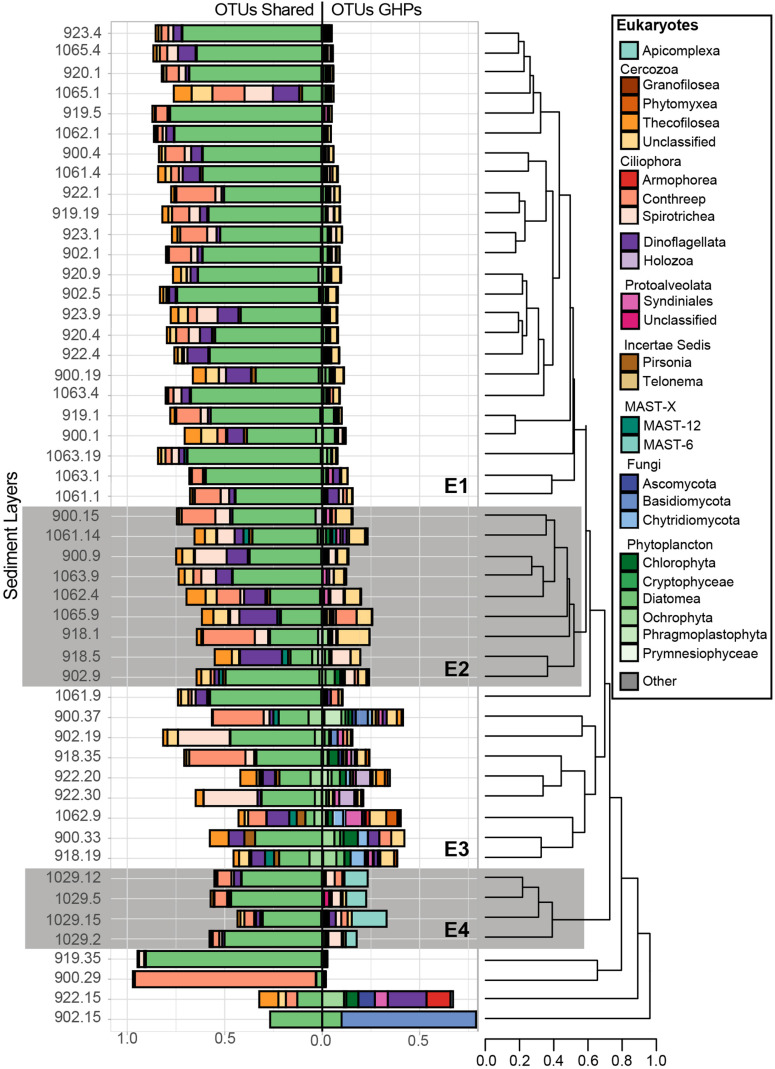
Relative abundance of the taxonomic groups that contain sequences associated to abundant eukaryotic OTUs. Overall, 140 abundant OTUs were retrieved, but 39 of them, abundant at the reference site, were mostly associated to taxa that are suggested to be allochthones and have fallen from surface waters. Therefore, for each sediment community library, these 39 OTUs were separated and are shown in the left bar charts (OTUs shared), while the remaining GHP OTUs are shown to the right (OTUs GHP). Bray-Curtis dissimilarity hierarchical clustering of the microbial communities at selected sediment depths was based on the GHP OTUs and separated these in four different clusters (E1, E2, E3, and E4). The eukaryotic communities from MC_902 at 15 cmbsf, from MC_900 at 29 cmbsf, from MC_919 at 35 cmbsf, and from MC_922 at 15 cmbsf strongly diverged and were therefore not included in these clusters. Leaves correspond to the core ID of the sediment layer and its depth [CoreID – depth (cm)]. “Other” corresponds to the relative abundance of sequences that were associated to taxonomic groups that are not illustrated in the figure.

For the bacterial domain, five community clusters, designated B1, B2, B3, B4, and B5, were identified for the GHP sediments (Figure 6). The rare biosphere represented by the non-abundant OTUs composed of a large fraction of all the bacterial communities and particularly for the clusters B1 and B2. Within these two clusters, the rare biosphere composed of an average of 76.7% of the bacterial sequences. Among the abundant OTUs, sequences within the cluster B1 were mostly assigned to the Gammaproteobacteria (5.2%), the Verrucomicrobiota (3.2%), and the Campilobacterota Sulfurimonadaceae (1.6%). The cluster B2 had stronger presence of Desulfobacterota Desulfobacterales (4.5%), including sequences associated to the cold seeps clade SEEP-SRB1 and Desulfobulbales (3.1%), in addition of Bacteroidota (4.7%). Cluster B3 represented sediment communities retrieved at the gas flare (core BC_1029) and was dominated by the Campilobacterota Sulfurovaceae (20.2%) and Sulfurimonadaceae (13.5%). Throughout all cores, the Sulfurovaceae and Sulfurimonadaceae were strictly represented by the genera *Sulfurovum* and *Sulfurimonas*, respectively. Additionally, B3 was characterized by the occurrence of Dissulfuribacterales (1.5%), mainly due to an OTU of the SEEP-SRB2 group, Desulfatiglandales (1.6%) and Desulfobacterales (2.9%). Remaining abundant taxa of the cluster B3 were assigned to the Bacteroidota (7.8%) and the Chloroflexi Anaerolinaeae (3.2%). It is also to be noted the presence of the Gammaproteobacteria Methylococcales in cluster B3 (1.2%). Communities within the cluster B4 primarily hosted sequences assigned to the Desulfobacterota (9.0%), largely included within the Desulfobacterales (5.2%), and the Chloroflexi Anaerolinaeae (6.1%) and Dehalococcoidia (3.6%). Additionally, abundant OTUs characterizing the cluster B4 were assigned to the Bacteroidota (4.3%), the Caldatribacteriota Japan Sea 1 (JS1) clade (4.8%), the Campilobacterota Sulfurimonadaceae (2.2%), and Sulfurovaceae (1.6%). The cluster B5 was dominated by the Desulfobacterota (19.6%), including representatives of Desulfobacterales (6.0%), Desulfatiglandales (4.4%), and Dissulfuribacterales (9.1%). One OTU assigned to SEEP-SRB2 and two OTUs assigned to SEEP-SRB1 composed 9.1 and 4.7% of the overall sequences, respectively (Figure 5). In comparison to other bacterial clusters, the cluster B5 was also characterized by a higher relative abundance of the Caldatribacteriota JS1 (10.7%) in addition to the Chloroflexi Dehalococcoidia (11.7%) and Anaerolinaeae (4.4%). Sediment samples clustering within the groups B3–B5 were mostly dominated by abundant OTUs, as the rare biosphere composed ca. 43% of the overall sequences.

With the eukaryotic primers, 39 abundant OTUs were retrieved at the reference core MC_898 and they composed from 20 to 100% of the sequences in all sediment communities. In Figure 7, these 39 OTUs are presented separately from the 101 eukaryotic OTUs retrieved exclusively at the GHPs. Beta diversity in the relative abundances of the taxonomic groups of these 101 OTUs retrieved in sediment communities at the GHPs site resulted in four clusters, designated as E1, E2, E3, and E4 (Figure 7). The proportions of sequences assigned to these OTUs varied between clusters, where an average of 8.5, 19.8, 29.8, and 24.1% of the sequences for the clusters E1, E2, E3, and E4 were assigned to them, respectively. In cluster E1, Cercozoa and ciliates corresponded respectively to 2.7 and 1.5% of the overall sequences. Within the cluster E2, these groups were more abundant, and their relative abundances increased to 8.2% for the Cercozoa and to 5.4% for the ciliates. For clusters B1 and B2, sequences were primarily assigned to an unclassified group of Cercozoa, while the class Spirotrichea primarily dominated the ciliates. Within the cluster E3, the taxonomic diversity was higher than for E1 or E2. Other cercozoan groups, such as Granofilosea, Phytomyxea, and Thecofilosea, in addition to the ciliates classes Armophorea and Conthreep, are frequently seen in higher abundances. In addition to Cercozoa and ciliates, abundant taxa exclusive to these sediment layers included representatives of the Holozoa, uncultivated marine stramenopiles (MAST) groups 6 and 12, in addition to the fungi (Ascomycota, Basidiomycota, and Chrytridiomycota). Sediment samples clustering within the cluster E4 were characterized by a higher proportion of Apicomplexa among the OTUs_._

Similarly to the distribution of the 101 eukaryotic OTUs presented above, the community structure of the 39 OTUs also thriving at the reference site varied between the clusters. The relative abundances of Ochrophyta were lower in clusters E2, E3, and E4, which are more predominantly composed by Cercozoa and ciliates. Finally, alpha diversity metrics that were used to assess biodiversity richness and evenness and the taxonomic composition for all domains within each sediment community are available as Supplementary Information (Supplementary Tables 8, 9 and Figures 1–3).

### Distribution and Co-occurrence of the Domain Clusters

The community clusters showed particular patterns of co-occurrence between each domain, especially for the prokaryotes (Supplementary Figure 4). For instance, 12 of the 13 sediments communities within cluster A1 were associated with the bacterial cluster B1. The pairs A2/B2, A3-A4/B4, and A6/B5 were also commonly co-occurring. However, concomitance patterns between prokaryotic and eukaryotic clusters were less supported. Still, the eukaryotic cluster E1 usually fell together with the pairs A1/B1 or A2/B2. The clusters E2 and E3, instead, coincided with the pairs A3-A4/B4 and A6/B5, respectively. The paired clusters A1/B1 were retrieved at the surface of nearly all sediment cores while the clusters A2/B2 generally corresponded to the subsurface communities at the reference site and at cores taken toward the edge of a GHP. Pairs of A3/B4 or A4/B4 occurred below the sediment surface at the apex of GHP 1 (cores MC_900 and MC_902) and of GHP 3 (MC_1061 and MC_1062). The pair A6/B5 occurred in subsurface sediments at the apex of GHP 1, but also toward the outskirt of the GHPs at the surface of MC_918 (GHP 1) and in subsurface sediments of GHP 5 (core MC_922). Finally, the microbial communities retrieved at the gas flare (core BC_1029) of the GHP 3 could not be related to other communities at the GHPs site for all domains of life and clustered separately. Communities from all sediment depths at BC_1029 clustered within A5, B3, and E4.

### Impact of Environmental Conditions on the Microbial Community Structure

**FIGURE 8:**
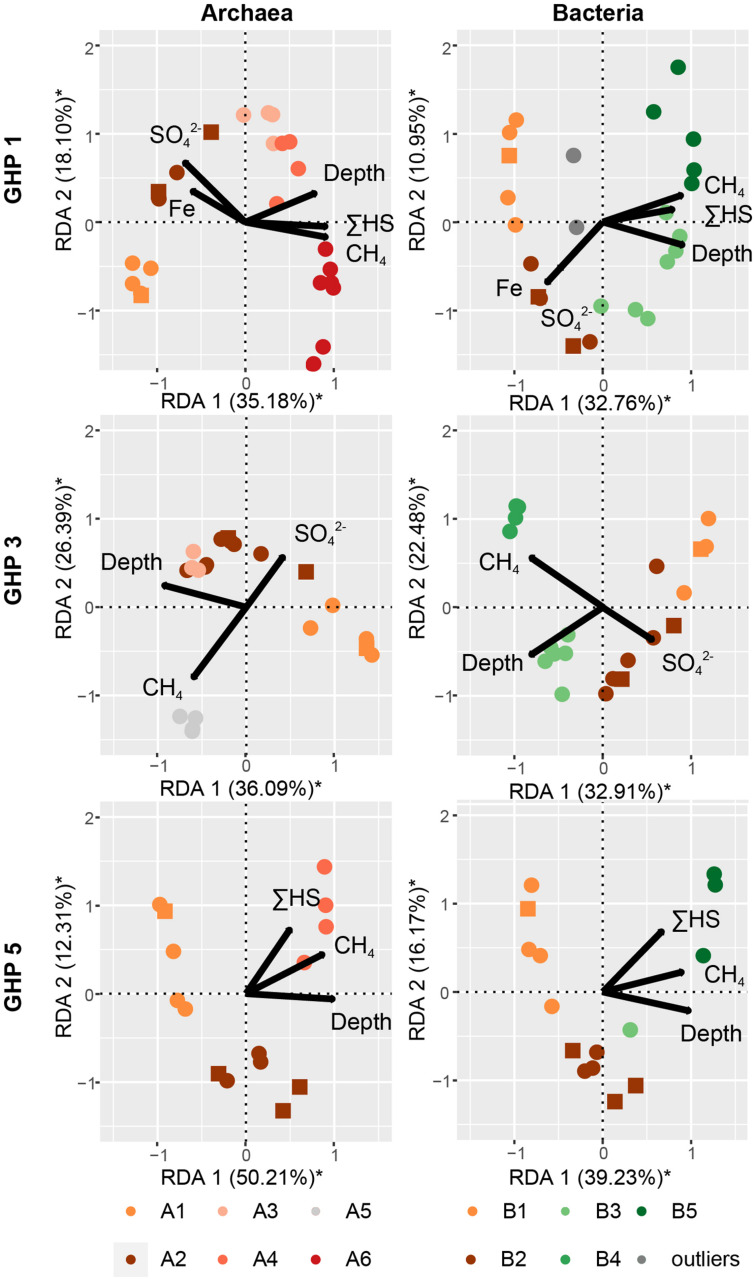
The impact of different environmental parameters on the archaeal and bacterial community structure within the sediments of the different GHPs assessed through dbRDA. A distance matrix was calculated based on the Bray-Curtis dissimilarity index from the composition of abundant archaeal and bacterial OTUs for each GHP. The correlation between the environmental variables and the built distance matrices are presented by biplots. The unconstrained proportion for each axis explaining the variability in a distance matrix is presented in percentage along the axis. Permutation tests were used to assess the solidity of the analyses and axes with a * were found significant.

The community clusters for the two prokaryotic domains demonstrated a profile primarily related to sediment depth and methane availability (Figure 8). The impact of measured environmental parameters on the dissimilarity between the different prokaryotic communities, observed through the formation of six archaeal and five bacterial community types, was assessed through dbRDA. Overall, the unconstrained proportions of the two principal axes (RDA 1 and 2) explained 43.71–62.52% of the dissimilarity between the different prokaryotic communities and were all significant (Figure 8). Depth correlated negatively with the prokaryotic community types A1 and B1 while CH_4_ concentrations drove the dissimilarity between the other community types. At all GHPs, A2 and B2 correlated negatively with CH_4_ concentrations, while A3-4-5-6 and B3-4-5 correlated positively. At GHP 1, these community types were also impacted by higher concentrations of ΣHS, while types A2 and B2 thrived in sediments richer in Fe^2+^ and SO_4_^2–^.

### Sulfurimonas and Agglutinated Foraminifera Relationship

In general, we found lower numbers of agglutinated foraminifera at habitats characterized by higher densities of the sulfide-oxidizing Suflurimonas. The relationship between the logarithm of the number of resampled *Sulfurimonas* sequences at the sediment surface and the density of agglutinated foraminiferal species showed a significant (*F* = 43.122, *p*-value = 0.007183), negative, and linear correlation (Supplementary Figure 5).

## Discussion

### Community Types Distribution Across the Pingos

Our first objective was to test the hypothesis that variations in the community structure occur along a radial gradient from the apex of the GHPs, which was expected to concentrate most of the gas seeping activity (Serov et al., 2017). Investigating the microbial communities thriving along spatial and depth pingos gradients led to the distinction of different community clusters for each domain of life (Figures 4, 6, 7). CH_4_-rich sediments hold distinct community clusters (A3–A6, B3–B5, E2–E4) while communities retrieved in CH_4_-poor sediments were more similar to the reference site (Figure 8). According to our hypothesis, CH_4_-rich sediments were recovered from coring locations close to the apex of GHP 1 (MC_900 and MC_902) or GHP 3 (BC_1029) where active gas flares were visible. However, we did also find high dissolved methane concentrations sediments hosting the CH_4_-rich community clusters we have described at the edge of GHPs 1 (MC_918) and 5 (MC_922; Supplementary Figure 4). This unpredicted spatial distribution of the different microbial community types at the GHPs was further supported through the observed significant negative correlation between the relative abundance of *Sulfurimonas* and the density of agglutinated foraminifera on the seafloor (Supplementary Figure 5). While *Sulfurimonas* is a genus that is often retrieved in higher relative abundances in CH_4_-rich sediments (Figure 6; Niemann et al., 2013; Bomberg et al., 2015), agglutinated foraminifera are known to be sensitive to CH_4_-rich environments (Panieri and Sen Gupta, 2008; Martin et al., 2010; Dessandier et al., 2019).

The use of these two independent methods further confirmed that there was no radial gradient at the GHPs. This contrasted thereby with earlier studies on active mud volcanoes where the community composition and the nature of the dominating methane oxidizers varied along concentric zones around the apex of the structure (Niemann et al., 2006; Lösekann et al., 2007; Lee et al., 2019). Instead, across the GHPs, community types were scarcely distributed and mainly depth and the availability of CH_4_ appeared to drive the transition between them (Figure 8). Furthermore, changes in community composition at the GHPs occurred on a smaller scale than at the HMMV, where the identified concentric zones extended over tenth to hundreds meters. In our study, nearby sediments cores MC_918 and MC_919, or BC_1029 and MC_1061, were less than 40 m apart, but the first hosted a community type dominated by ANME-1 while the latter was more similar to the reference site. This suggests that the microbial community spatial succession at these pingos is still not yet fully grasped. Thereby, further investigations on the variability of the microbial community composition should be addressed at a higher site resolution.

### Microbial Biodiversity Across the Study Area

Our second objective was to describe the microbial biodiversity at the GHPs and to identify key taxa influenced by this CH_4_-rich environment. Overall, the communities presented different assemblages, depending on their vertical positioning in the sediment matrix; i.e., surface, a few cm below the seafloor, in CH_4_-rich sediments, or at the gas flare (BC_1029). The variability in the structure of eukaryotic communities and the nature and quantities of Foraminifera at the GHPs were analyzed differently than for prokaryotes. We therefore discuss the composition of the prokaryotic and eukaryotic communities within the different sediment habitats separately.

#### Prokaryotes

Sediment characterized by a CH_4_ depletion and ΣHS increase hosted a microbial community dominated by ANME and SRBs, strongly suggesting ongoing AOM. The archaeal community was primarily dominated across all GHPs by the anaerobic CH_4_ oxidizing group ANME-1 (Figures 4, 5). Interestingly, methanotrophic communities primarily driven by ANME-1 have been less frequently observed than by ANME-2, or were found only in deeper sediments (Girguis et al., 2005; Ruff et al., 2015; Gründger et al., 2019). Our understanding of the factors favoring the growth of the different ANME groups is still limited. Their tolerance levels to various environmental factors and the impact of CH_4_ flux rates on their growth rate have been two common orientations used by studies to investigate their biogeography. Within the first orientation, it is suggested that the ANME-1 would be more tolerant to broader ranges of environmental conditions, and could predominate over ANME-2 in low SO_4_^2–^ and high HS^–^ environments (Timmers et al., 2015). These different tolerances to the presence of SO_4_^2–^ and HS^–^ has been suggested to explain vertical successions in dominance of these groups along different SMTZ (Roalkvam et al., 2011; Biddle et al., 2012; Ruff et al., 2015). However, at the GHPs, although ANME-2 and ANME-3 were also detected, their relative abundances remained low, and there was no clear vertical transition in the nature of the dominant ANME group along the SMTZ in cores MC_900 and MC_902. This could suggest that other factors at the GHPs favor the growth of ANME-1 and/or inhibit the proliferation of ANME-2. Within the second orientation, observations were made at the Hydrate Ridge or the Gulf of Mexico that ANME-2 groups were more commonly retrieved in areas with highly active CH_4_ seepage (Vigneron et al., 2013, 2019). In our study, although ANME-1 still predominated the methanotrophic community near the gas flare (BC_1029), the relative abundances of ANME-2 groups were in contrast higher than in other clusters. However, this hypothesis would contradict previous observations where ANME-2 demonstrated higher growth rates than ANME-1 at low CH_4_ flux rates (Girguis et al., 2005). Beyond these two hypotheses presented above, the hydrographic conditions above the GHPs could also induce an additional set of environmental constraints, as the bottom-water temperature seasonally varies (Ferré et al., 2020). This creates fluctuations in both CH_4_ seeping activity from the sediments and subsequently CH_4_ oxidation rates in the water column. This seasonality in CH_4_ seepage activity could potentially also impact the selection of the ANME groups. The biogeography of ANME groups remains therefore still unclear. With its five GHPs presenting different CH_4_ flux history and its multiple ecological niches, the GHPs, combined with the usage of appropriate tools for sampling sediments at a higher precision, present thereby an ideal site to provide further insights into the distribution of ANME groups.

Furthermore, to mediate AOM, ANME groups require an electron acceptor, such as sulfate, and have therefore been frequently observed in consortia with microorganisms capable of reducing these compounds. The ANME-1 group have regularly been assigned to the uncultured groups of SEEP-SRB1 and SEEP-SRB2, where both are detected in CH_4_-rich sediments at the GHPs. In our study, the relative abundance of Desulfobacterota was higher in microbial communities dominated by ANME groups (Figure 6). Furthermore, the decreasing concentration of SO_4_^2–^ with depth in CH_4_-rich sediments, combined with an increasing availability of ΣHS, strongly suggested the use of sulfate as the electron acceptor for AOM. However, across all the GHPs, there was no positive correlation between the relative abundance of ANME-1 and a particular SRB group, either SEEP-SRB 1 or 2, further supporting the hypothesis that ANME-1 could metabolize CH_4_ alone (Figures 4–6). Indeed, it was observed that ANME-1 could perform both AOM and sulfate reduction within the same cell (Milucka et al., 2012) and the detection of F420-dependent sulfite reductase in ANME-1 communities may be part of this novel pathway (Vigneron et al., 2019). Nevertheless, a previous study could not find a correlation of ANME-1 and the abundance of dissimilatory sulfite reductase, an essential enzyme for active SRB (Vigneron et al., 2019), demonstrating that ANME-1 may not be able to perform SR. Finally, a different explanation of the absent correlation between ANME-1 and SRB groups could be due to the usage of intercellular wires forming cell-to-cell connections for electron transfers, a hypothesis supported by the detection of genes expressing for extracellular cytochrome production, between distanced ANME-1 and SRB cells (Wegener et al., 2015). Our results, based on the sulfate and sulfide profiles, advocate an anaerobic oxidation of CH_4_ supported by the reduction of sulfate, but the role of Desulfobacterota and its relation with the ANME groups remain unclear.

While AOM is mediated by ANME in anaerobic environment, methanotrophy in an aerobic environment is primarily performed by distinct bacterial groups (Hanson and Hanson, 1996; Knief, 2015). In our study, higher concentrations of CH_4_ than at the reference site were detected at the surface of some sediment cores collected at the GHPs. However, despite the availability of oxygen suggested by the presence of aerobic taxonomic groups, aerobic bacterial methanotrophs were barely detected. We retrieved abundant Verrucomicrobiales OTUs at the surface of most sediment cores, but their assigned family Rubritaleaceae is not known to include aerobic methanotrophs. Aerobic methanotrophs (Methyloccocales) could only be detected at the surface of BC_1029, collected near the gas flare, but this OTU was composed of only 1.2% of all bacterial sequences. Surprisingly, the apparent rarity of aerobic CH_4_ oxidizers is contrasting to most seep sites where they were found when both CH_4_ and O_2_ are present (Lösekann et al., 2007; Roalkvam et al., 2011; Ruff et al., 2015). Nevertheless, we cannot disregard that the near absence of aerobic methanotrophs in our amplicon libraries could be caused by the choice of primers used (McDonald et al., 2008). Different approaches, including the use of primers targeting functional genes such as *pmoA*, would be required to improve the study of the biodiversity of aerobic methane oxidizers. Finally, CH_4_-rich sediments also harbored higher relative abundances of other groups, but which are likely not directly involved in the AOM. Chloroflexi, the Caldatribacteriota JS1, and Campilobacterota groups were also in higher abundance in CH_4_-rich sediments than at other sediment layers. Similarly to the distribution of ANME groups, these bacterial groups showed different relative abundances between CH_4_-rich sediments collected at the gas flare to the other samples. While most communities in CH_4_-rich sediments demonstrated high proportions of Chloroflexi and JS1, the bacterial communities at the gas flare was primarily dominated by sulfide oxidizing bacteria (Figure 6). More precisely, two Campilobacterota genera mediating the oxidation of sulfur, sulfide or thiosulfate, *Sulfurimonas* and *Sulfuvorum*, were found in abundance. These genera are commonly found in abundance near hydrothermal plumes and in diffusive flow sediments, as well as at cold seeps (Yamamoto and Takai, 2011; Adams et al., 2013), while sulfide oxidization in marine sediments tends to be driven primarily by Alphaproteobacteria or Gamma proteobacteria (Lenk et al., 2011). In our study, similar observations suggest that these bacteria play an important role in sulfur cycling and largely dominated the bacterial communities at the gas flare, in comparison to the other sites.

In the absence of CH_4_, the sediment microbial composition at the GHPs was highly similar to the reference site and was primarily driven by depth (Figure 8). Depth is likely influencing the shape of microbial communities at the GHPs through the presence or absence of oxygen, a parameter well-known to shape the structure of microbial communities in sediments (Fenchel and Finlay, 2008). Surface sediments were primarily dominated by the aerobic ammonia-oxidizing archaea (AOA) Nitrosopumilaceae that plays, along with ammonia-oxidizing bacteria, an important role in the transformation of nitrogen compounds in marine systems, including cold seeps or at hydrothermal vents (Könneke et al., 2005; Dang et al., 2009; Miyazaki et al., 2009; Stahl and de la Torre, 2012). In deeper sediments, the archaeal community (A2) was dominated by Woesearchaeales and Bathyarchaeia (Figure 4). The most abundant OTU of the 38 associated to the Woeserchaeia across all clusters was found predominantly at nearly all sediment layers below the seafloor, including in the CH_4_-rich sediments. As oxygen availability is suggested to be the main factor determining the nature of the thriving Wosearchaeales (Liu et al., 2018), its detection in deeper sediments likely suggest an anoxic ecotype that may be involved in a fermentation-based lifestyle (Castelle et al., 2015). Bathyarcheia, previously known as the Miscellaneous Crenarchaeotal Group (MCG), and the thermoplasmatales MBG-D are globally abundant in marine sediments. The detection of protein-degrading enzymes suggest a role in organic matter anaerobic degradation (Webster et al., 2010; Kubo et al., 2012; Lloyd et al., 2013). The relative abundance of OTUs assigned to the Desulfobacterota within the bacterial community increased with depth, but remained lower than in CH_4_-rich sediments (cluster B5). The presence of these Desulfobacterota groups are common in marine sediments as they play a major role in mineralizing organic matter through sulfate reduction (Jørgensen, 1982; Abu Laban et al., 2015; Robador et al., 2016).

#### Eukaryotes

Microbial eukaryotic communities at cold seeps have received less attention than the prokaryotes, despite their active role as part of bacterial mat type habitats for instance, or the capacity of some to harbor sulfur oxidizing bacteria (Buck and Barry, 1998; Buck et al., 2000). In this study, we investigated protists and fungi based on the V4 region of the 18S rDNA and have identified key taxonomic groups thriving at the GHP sites. Across all sediments layers, large fractions of sequences that clustered into OTUs were assigned to Metazoa and to sedimenting allochthonous cells. The removal of these sequences likely affected the following analyses of the GHPs eukaryotic communities. Therefore, we assessed separately the 39 OTUs proliferating at the reference site from the 101 OTUs found in abundance only at the GHPs to highlight eukaryotic taxa thriving in CH_4_-rich sediments. Communities clustering in E1 demonstrated high similarity to the reference site and could be retrieved at different distances from the apex of all GHPs, but were limited to sediments characterized by low CH_4_ concentrations. We thereby demonstrated that in the absence of CH_4_, eukaryotic communities across the GHPs have similar composition than to the reference site. In contrast, clusters E2–E4 were retrieved in or near CH_4_-rich sediments and demonstrated higher relative abundances of OTUs that are absent or barely found at the reference site.

Within the cluster E2, these OTUs were primarily assigned to ciliates and Cercozoa. We also noted that within the 39 subtracted OTUs, the fraction of alveolates and Cercozoa increased and even surpassed their relative abundances in E3 and E4. Higher densities of prokaryotes involved directly or indirectly in AOM can be a food source for these potential heterotrophic eukaryotes, but their growth in communities clustering in E3 and E4 may be limited by the toxicity of sulfidic conditions (Massana et al., 1994; Coyne et al., 2013). Communities clustering in E3 occurred primarily in CH_4_-rich sediments with the prokaryotic communities of clusters A4-A6/B5, composed of taxa involved in AOM and sulfate reduction (Supplementary Figure 4). Nearly all communities within the cluster E3 hosted the highest relative abundances of sequences associated to the 101 OTUs that are exclusively found in abundance at the GHPs (Figure 7). The contrast in these relative abundances, in comparison to the cluster E1, demonstrates the impact of CH_4_ on the eukaryotic diversity. The assignment of these OTUs was strongly heterogeneous as several taxonomic groups, such as the Protoalveolata Syndiniales, were present only in few communities (Figure 7). The eukaryotic communities within this cluster were also characterized by the emergence of fungal taxonomic groups. Communities within the cluster E3 were especially affected by the proportion of sequences associated to Metazoa, as on average 40% of the sequences were assigned to this taxon and had to be removed. Thereby, while the heterogeneity in the structure of the communities clustering in E3 could be caused by local conditions, we cannot rule out that it may be due to limitations in the coverage of the eukaryotic biodiversity. Communities at the gas flare (BC_1029), similarly as for the prokaryotes, hosted a distinctive eukaryotic biodiversity clustering exclusively in E4. Among the sequences assigned to the OTUs exclusively abundant at the GHPs within E4, most were primarily assigned to Apicomplexa (up to 15%). Apicomplexa are parasitic alveolates, but the nature of potential hosts at the gas flare remains unknown. Overall, our results demonstrated that changes in the eukaryotic biodiversity occur in CH_4_-rich sediments. Using different approaches, such as targeting specific genes or using blocking primers, may provide a more accurate profile of eukaryotic biodiversity at the GHPs. These investigations would further improve our understanding on the role of these protists and fungi at the GHPs site on the microbial community, the biogeochemical cycles, and on food web structures.

Overall, our approach suggests that CH_4_ and oxygen are two key factors influencing the microbial community structure. Nevertheless, communities within a cluster had up to approximately 60% similarity and the dendrograms (Figures 4, 6, 7) present additional sub-clusters at higher thresholds. It advocates therefore for additional factors influencing the distribution patterns of the microbial taxonomic groups at the GHPs site. Thereby, our study revealed that the GHP ecosystem has to be considered in further investigations as a myriad of ecological niches. In this perspective, the distance between the cores (approx. 20 m) at a GHP is likely too long to investigate gradual changes in microbial communities in relation to fluxes of CH_4_. Designing an approach at a small scale may better fill these gaps of knowledge.

## Summary and Concluding Remarks

This study shows that both prokaryotic and eukaryotic communities at the GHPs formed a unique structure influenced by the complex distribution of CH_4_ seepage. The distribution of the community types presented similar chaotic patterns and methane oxidizing communities could be retrieved at different locations over a GHP. In CH_4_-rich sediments, AOM seemed to be primarily driven by a single OTU associated to ANME-1 and had no correlation with a group of SRB. This further supports the hypotheses that ANME-1 can mediate AOM alone or use different sources of electron receptors. Our approach also illustrated that at the GHPs site, metabolites of AOM, such as sulfide and organic compounds, likely explain the predominance of additional taxa, including the Campilobacterota, the thermoplasmatales MBG-D, and the Bathyarchaeia. Eukaryotic communities in the CH_4_-rich sediments had a dominance of heterotrophic ciliates and Cercozoa, likely benefiting from the higher abundances of prokaryotes as a food source. The retrieval of these taxa, distributed specifically among the GHPs, suggests a complex functional microbial system supported by, or contributing to, the local oxidation of CH_4_.

## Data Availability Statement

The datasets generated for this study can be found in the Sequence Read Archive Genebank as BioProject accession number PRJNA593930.

## Author Contributions

VC, MS, FG, and HN initially designed the project. VC, FG, MS, HN, and P-AD contributed to the sampling. VC and P-AD performed the laboratory manipulations, sequences analyses, and statistics with advice from DK, MS, FG, and HN. VC wrote the manuscript with input from DK, MS, HN, FG, P-AD, and GP. All authors contributed to the article and approved the submitted version.

## Conflict of Interest

The authors declare that the research was conducted in the absence of any commercial or financial relationships that could be construed as a potential conflict of interest.

## Footnotes

1 https://cage.uit.no/wp-content/uploads/2019/02/15-2.cage-cruise-report-public-1.pdf

2 https://cage.uit.no/wp-content/uploads/2019/02/16-5.cage-cruise-report-public.pdf

3 https://cage.uit.no/wp-content/uploads/2019/02/17-2.cage-cruise-report-public.pdf

4 http://www.ncbi.nlm.nih.gov/sra

5 http://drive5.com/usearch/manual/uparse_pipeline.html

## References

[B1] Abu Laban N. Tan B. Dao A. Foght J. (2015). Draft Genome Sequence of Uncultivated Toluene-Degrading Desulfobulbaceae Bacterium Tol-SR, Obtained by Stable Isotope Probing Using [13C6] Toluene. *Genom. Announc.* 3 e1423–e1414. 10.1128/genomeA.01423-1414 PMC4299903 25593261

[B2] Adams M. M. Hoarfrost A. L. Bose A. Joye S. B. Girguis P. R. (2013). Anaerobic oxidation of short-chain alkanes in hydrothermal sediments: potential influences on sulfur cycling and microbial diversity. *Front. Microbiol.* 4:110. 10.3389/fmicb.2013.00110 23717305 PMC3653109

[B3] Åström E. K. L. Carroll M. L. Ambrose W. G. Sen A. Silyakova A. Carroll J. (2018). Methane cold seeps as biological oases in the high-Arctic deep sea. *Limnol. Oceanogr.* 63 S209–S231. 10.1002/lno.10732

[B4] Atkins M. Hanna M. Kupetsky E. Saito M. Taylor C. Wirsen C. (2002). Tolerance of flagellated protists to high sulfide and metal concentrations potentially encountered at deep-sea hydrothermal vent. *Mar. Ecol. Prog. Ser.* 226 63–75. 10.3354/meps226063

[B5] Beal E. J. House C. H. Orphan V. J. (2009). Manganese- and iron-dependent marine methane oxidation. *Science* 325 184–187. 10.1126/science.1169984 19589998

[B6] Bhattarai S. Cassarini C. Rene E. R. Zhang Y. Esposito G. Lens P. N. L. (2018). Enrichment of sulfate reducing anaerobic methane oxidizing community dominated by ANME-1 from Ginsburg Mud Volcano (Gulf of Cadiz) sediment in a biotrickling filter. *Bioresour. Technol.* 259 433–441. 10.1016/j.biortech.2018.03.018 29602106

[B7] Biddle J. F. Cardman Z. Mendlovitz H. Albert D. B. Lloyd K. G. Boetius A. (2012). Anaerobic oxidation of methane at different temperature regimes in Guaymas Basin hydrothermal sediments. *ISME J.* 6 1018–1031. 10.1038/ismej.2011.164 22094346 PMC3329104

[B8] Boetius A. Ravenschlag K. Schubert C. J. Rickert D. Widdel F. Gieseke A. (2000). A marine microbial consortium apparently mediating anaerobic oxidation of methane. *Nature* 407 623–626. 10.1038/35036572 11034209

[B9] Boetius A. Wenzhöfer F. (2013). Seafloor oxygen consumption fuelled by methane from cold seeps. *Nat. Geosci.* 6 725–734. 10.1038/ngeo1926

[B10] Bomberg M. Nyyssönen M. Pitkänen P. Lehtinen A. Itävaara M. (2015). Active Microbial Communities Inhabit Sulphate-Methane Interphase in Deep Bedrock Fracture Fluids in Olkiluoto. *Finland. Biomed Res. Int.* 2015:979530. 10.1155/2015/979530 26425566 PMC4573625

[B11] Boyce R. E. Edgar J. B. Saunders N. T. (1973). “Appendix I. Physical Properties - Methods,” in *Initial Reports of the Deep Sea Drilling Project*, Edn, 15 (United Kingdom: U.S. Government Printing Office), 1115–1128.

[B12] Buck K. R. Barry J. P. (1998). Monterey Bay cold seep infauna: Quantitative comparison of bacterial mat meiofauna with non-seep control sites. *Cah. Biol. Mar.* 39 333–335.

[B13] Buck K. R. Barry J. P. Simpson A. G. B. (2000). Monterey Bay cold seep biota: Euglenozoa with chemoautotrophic bacterial epibionts. *Eur. J. Protistol.* 36 117–126. 10.1016/S0932-4739(00)8002980022

[B14] Castelle C. J. Wrighton K. C. Thomas B. C. Hug L. A. Brown C. T. Wilkins M. J. (2015). Genomic expansion of domain archaea highlights roles for organisms from new phyla in anaerobic carbon cycling. *Curr. Biol.* 25 690–701. 10.1016/j.cub.2015.01.014 25702576

[B15] Cline J. D. (1969). Spectrophotometric determination of hydrogen sulfide in natural waters. *Limnol. Oceanogr.* 14 454–458. 10.4319/lo.1969.14.3.0454

[B16] Cordes E. E. Cunha M. R. Galéron J. Mora C. Olu-Le Roy K. Sibuet M. (2010). The influence of geological, geochemical, and biogenic habitat heterogeneity on seep biodiversity. *Mar. Ecol.* 31 51–65. 10.1111/j.1439-0485.2009.00334.x

[B17] Corliss B. H. (1991). Morphology and microhabitat preferences of benthic foraminifera from the northwest Atlantic Ocean. *Mar. Micropaleontol.* 17 195–236. 10.1016/0377-8398(91)90014-W

[B18] Coyne K. J. Countway P. D. Pilditch C. A. Lee C. K. Caron D. A. Cary S. C. (2013). Diversity and Distributional Patterns of Ciliates in Guaymas Basin Hydrothermal Vent Sediments. *J. Eukaryot. Microbiol.* 60 433–447. 10.1111/jeu.12051 23750565

[B19] Dang H. Li J. Zhang X. Li T. Tian F. Jin W. (2009). Diversity and spatial distribution of amoA -encoding archaea in the deep-sea sediments of the tropical West Pacific Continental Margin. *J. Appl. Microbiol.* 106 1482–1493. 10.1111/j.1365-2672.2008.04109.x 19187134

[B20] Daniel J. Fornari D. J. Group W. T. (2003). A new deep-sea towed digital camera and multi-rock coring system. *Eos Trans. Am. Geophys. Union* 84 69–73. 10.1029/2003EO080001

[B21] Dessandier P. A. Borrelli C. Kalenitchenko D. Panieri G. (2019). Benthic Foraminifera in Arctic Methane Hydrate Bearing Sediments. *Front. Mar. Sci.* 2019:6 10.3389/fmars.2019.00765

[B22] Edgar R. C. (2010). Search and clustering orders of magnitude faster than BLAST. *Bioinformatics* 26 2460–2461. 10.1093/bioinformatics/btq461 20709691

[B23] Ettwig K. F. Butler M. K. Le Paslier D. Pelletier E. Mangenot S. Kuypers M. M. M. (2010). Nitrite-driven anaerobic methane oxidation by oxygenic bacteria. *Nature* 464 543–548. 10.1038/nature08883 20336137

[B24] Ettwig K. F. Zhu B. Speth D. Keltjens J. T. Jetten M. S. M. Kartal B. (2016). Archaea catalyze iron-dependent anaerobic oxidation of methane. *Proc. Natl. Acad. Sci.* 113 12792–12796. 10.1073/PNAS.1609534113 27791118 PMC5111651

[B25] Fenchel T. Finlay B. (2008). Oxygen and the Spatial Structure of Microbial Communities. *Biol. Rev.* 83 553–569. 10.1111/j.1469-185X.2008.00054.x 18823390

[B26] Ferré B. Jansson P. G. Moser M. Serov P. Portnov A. Graves C. A. (2020). Reduced methane seepage from Arctic sediments during cold bottom-water conditions. *Nat. Geosci.* 13 144–148. 10.1038/s41561-019-0515513

[B27] Girguis P. R. Cozen A. E. DeLong E. F. (2005). Growth and population dynamics of anaerobic methane-oxidizing archaea and sulfate-reducing bacteria in a continuous-flow bioreactor. *Appl. Environ. Microbiol.* 71 3725–3733. 10.1128/AEM.71.7.3725-3733.2005 16000782 PMC1169053

[B28] Gründger F. Carrier V. Svenning M. M. Panieri G. Vonnahme T. R. Klasek S. (2019). Methane-fuelled biofilms predominantly composed of methanotrophic ANME-1 in Arctic gas hydrate-related sediments. *Sci. Rep.* 9:9725 10.1038/s41598-019-4620946205 PMC6611871 31278352

[B29] Hanson R. S. Hanson T. E. (1996). Methanotrophic Bacteria. *Microbiol. Rev* 60 439–471. 10.1128/mmbr.60.2.439-471.1996 8801441 PMC239451

[B30] Hoehler T. M. Borowski W. S. Alperin M. J. Rodriguez N. M. Paull C. K. (2000). “Model, stable isotope, and radiotracer characterization of anaerobic methane oxidation in gas hydrate-bearing sediments of the Blake Ridge,” in *Proceedings of the Ocean Drilling Program: Scientific Results*, 79–85 (Texas: College Station). 10.2973/odp.proc.sr.164.242.2000

[B31] Hong W. L. Latour P. Sauer S. Sen A. Gilhooly W. P. Lepland A. (2020). Iron cycling in Arctic methane seeps. *Geo Mar. Lett.* 40 1–11. 10.1007/s00367-020-00649645

[B32] Hong W. L. Torres M. E. Carroll J. Crémière A. Panieri G. Yao H. (2017). Seepage from an arctic shallow marine gas hydrate reservoir is insensitive to momentary ocean warming. *Nat. Commun.* 8 1–14. 10.1038/ncomms15745 28589962 PMC5477557

[B33] Hong W.-L. Torres M. E. Portnov A. Waage M. Haley B. Lepland A. (2018). Variations in Gas and Water Pulses at an Arctic Seep: Fluid Sources and Methane Transport. *Geophys. Res. Lett.* 45 4153–4162. 10.1029/2018GL077309

[B34] Hovland M. Svensen H. (2006). Submarine pingoes: Indicators of shallow gas hydrates in a pockmark at Nyegga, Norwegian Sea. *Mar. Geol.* 228 15–23. 10.1016/J.MARGEO.2005.12.005

[B35] Hu B. Shen L. Lian X. Zhu Q. Liu S. Huang Q. (2014). Evidence for nitrite-dependent anaerobic methane oxidation as a previously overlooked microbial methane sink in wetlands. *Proc. Natl. Acad. Sci. U. S. A.* 111 4495–4500. 10.1073/pnas.1318393111 24616523 PMC3970540

[B36] Jacobsen C. S. Nielsen T. K. Vester J. K. Stougaard P. Nielsen J. L. Voriskova J. (2018). Inter-laboratory testing of the effect of DNA blocking reagent G2 on DNA extraction from low-biomass clay samples. *Sci. Rep.* 8:5711. 10.1038/s41598-018-24082-y 29632323 PMC5890260

[B37] James R. H. Bousquet P. Bussmann I. Haeckel M. Kipfer R. Leifer I. (2016). Effects of climate change on methane emissions from seafloor sediments in the Arctic Ocean: A review. *Limnol. Oceanogr.* 61 S283–S299. 10.1002/lno.10307

[B38] Jernas P. Klitgaard-Kristensen D. Husum K. Koç N. Tverberg V. Loubere P. (2018). Annual changes in Arctic fjord environment and modern benthic foraminiferal fauna: Evidence from Kongsfjorden, Svalbard. *Glob. Planet. Change* 163 119–140. 10.1016/j.gloplacha.2017.11.013

[B39] Jørgensen B. B. (1982). Mineralization of organic matter in the sea bed—the role of sulphate reduction. *Nature* 296 643–645. 10.1038/296643a0

[B40] Joye S. B. Bowles M. W. Samarkin V. A. Hunter K. S. Niemann H. (2010). Biogeochemical signatures and microbial activity of different cold-seep habitats along the Gulf of Mexico deep slope. *Deep Sea Res. Part II Top. Stud. Oceanogr.* 57 1990–2001. 10.1016/J.DSR2.2010.06.001

[B41] Knief C. (2015). Diversity and habitat preferences of cultivated and uncultivated aerobic methanotrophic bacteria evaluated based on pmoA as molecular marker. *Front. Microbiol.* 6:1346. 10.3389/fmicb.2015.01346 26696968 PMC4678205

[B42] Knittel K. Boetius A. (2009). Anaerobic Oxidation of Methane: Progress with an Unknown Process. *Annu. Rev. Microbiol.* 63 311–334. 10.1146/annurev.micro.61.080706.093130 19575572

[B43] Knittel K. Boetius A. Lemke A. Eilers H. Lochte K. Pfannkuche O. (2003). Activity, distribution, and diversity of sulfate reducers and other bacteria in sediments above gas hydrate (Cascadia margin. *Oregon)*. *Geomicrobiol. J.* 20 269–294. 10.1080/01490450303896

[B44] Knittel K. Lösekann T. Boetius A. Kort R. Amann R. (2005). Diversity and distribution of methanotrophic archaea at cold seeps. *Appl. Environ. Microbiol.* 71 467–479. 10.1128/AEM.71.1.467-479.2005 15640223 PMC544223

[B45] Koch S. Berndt C. Bialas J. Haeckel M. Crutchley G. Papenberg C. (2015). Gas-controlled seafloor doming. *Geology* 43 571–574. 10.1130/G36596.1

[B46] Könneke M. Bernhard A. E. de la Torre J. R. Walker C. B. Waterbury J. B. Stahl D. A. (2005). Isolation of an autotrophic ammonia-oxidizing marine archaeon. *Nature* 437 543–546. 10.1038/nature03911 16177789

[B47] Krüger M. Treude T. Wolters H. Nauhaus K. Boetius A. (2005). Microbial methane turnover in different marine habitats. *Palaeogeogr. Palaeoclimatol. Palaeoecol.* 227 6–17. 10.1016/j.palaeo.2005.04.031

[B48] Kubo K. Lloyd K. G. Biddle J. F. Amann R. Teske A. Knittel K. (2012). Archaea of the Miscellaneous Crenarchaeotal Group are abundant, diverse and widespread in marine sediments. *ISME J.* 6 1949–1965. 10.1038/ismej.2012.37 22551871 PMC3449235

[B49] Lee D. H. Lee Y. M. Kim J. H. Jin Y. K. Paull C. Niemann H. (2019). Discriminative biogeochemical signatures of methanotrophs in different chemosynthetic habitats at an active mud volcano in the Canadian Beaufort Sea. *Sci. Rep.* 9 1–13. 10.1038/s41598-019-5395053954 31772218 PMC6879587

[B50] Legendre P. Legendre L. (1998). *Numerical Ecology*, 2nd Edn Netherland: Elsevier Science, 24.

[B51] Lenk S. Arnds J. Zerjatke K. Musat N. Amann R. Mußmann M. (2011). Novel groups of Gammaproteobacteria catalyse sulfur oxidation and carbon fixation in a coastal, intertidal sediment. *Environ. Microbiol.* 13 758–774. 10.1111/j.1462-2920.2010.02380.x 21134098

[B52] Liu X. Li M. Castelle C. J. Probst A. J. Zhou Z. Pan J. (2018). Insights into the ecology, evolution, and metabolism of the widespread Woesearchaeotal lineages. *Microbiome* 6:102 10.1186/s40168-018-0488482 PMC5994134 29884244

[B53] Lloyd K. G. Schreiber L. Petersen D. G. Kjeldsen K. U. Lever M. A. Steen A. D. (2013). Predominant archaea in marine sediments degrade detrital proteins. *Nature* 496 215–218. 10.1038/nature12033 23535597

[B54] Lösekann T. Knittel K. Nadalig T. Fuchs B. Niemann H. Boetius A. (2007). Diversity and abundance of aerobic and anaerobic methane oxidizers at the Haakon Mosby Mud Volcano, Barents Sea. *Appl. Environ. Microbiol.* 73 3348–3362. 10.1128/AEM.0001617 17369343 PMC1907091

[B55] Mackay J. R. (1998). Pingo growth and collapse, Tuktoyaktuk Peninsula area, western arctic coast, Canada: A long-term field study. *Geogr. Phys. Quat.* 52 271–323. 10.7202/004847ar

[B56] Maignien L. Parkes R. J. Cragg B. Niemann H. Knittel K. Coulon S. (2013). Anaerobic oxidation of methane in hypersaline cold seep sediments. *FEMS Microbiol. Ecol.* 83 214–231. 10.1111/j.1574-6941.2012.01466.x 22882187

[B57] Marlow J. J. Steele J. A. Case D. H. Connon S. A. Levin L. A. Orphan V. J. (2014). Microbial abundance and diversity patterns associated with sediments and carbonates from the methane seep environments of Hydrate Ridge, OR. *Front. Mar. Sci.* 1:1–16. 10.3389/fmars.2014.00044

[B58] Martin R. A. Nesbitt E. A. Campbell K. A. (2010). The effects of anaerobic methane oxidation on benthic foraminiferal assemblages and stable isotopes on the Hikurangi Margin of eastern New Zealand. *Mar. Geol.* 272 270–284. 10.1016/j.margeo.2009.03.024

[B59] Massana R. Stumm C. K. Pedros-Alio C. (1994). Effects of temperature, sulfide, and food abundance on growth and feeding of anaerobic ciliates. *Appl. Environ. Microbiol.* 60 1317–1324. 10.1128/aem.60.4.1317-1324.1994 16349238 PMC201476

[B60] McDonald I. R. Bodrossy L. Chen Y. Murrell J. C. (2008). Molecular ecology techniques for the study of aerobic methanotrophs. *Appl. Environ. Microbiol.* 74 1305–1315. 10.1128/AEM.022332237 18165358 PMC2258629

[B61] Milucka J. Ferdelman T. G. Polerecky L. Franzke D. Wegener G. Schmid M. (2012). Zero-valent sulphur is a key intermediate in marine methane oxidation. *Nature* 491 541–546. 10.1038/nature11656 23135396

[B62] Miyazaki J. Higa R. Toki T. Ashi J. Tsunogai U. Nunoura T. (2009). Molecular characterization of potential nitrogen fixation by anaerobic methane-oxidizing archaea in the methane seep sediments at the number 8 Kumano Knoll in the Kumano Basin, offshore of Japan. *Appl. Environ. Microbiol.* 75 7153–7162. 10.1128/AEM.011841189 19783748 PMC2786543

[B63] Nauhaus K. Treude T. Boetius A. Kruger M. (2005). Environmental regulation of the anaerobic oxidation of methane: a comparison of ANME-I and ANME-II communities. *Environ. Microbiol.* 7 98–106. 10.1111/j.1462-2920.2004.00669.x 15643940

[B64] Niemann H. Elvert M. Hovland M. Orcutt B. Judd A. Suck I. (2005). *Methane emission and consumption at a North Sea gas seep (Tommeliten area). Biogeosciences Discuss. 2.* Available at: https://hal.archives-ouvertes.fr/hal-00297796 [accessed January 31, 2020].

[B65] Niemann H. Fischer D. Graffe D. Knittel K. Montiel A. Heilmayer O. (2009). Biogeochemistry of a low-activity cold seep in the Larsen B area, western Weddell Sea. *Antar. Biogeosci.* 6 2383–2395. 10.5194/bg-6-23832009

[B66] Niemann H. Linke P. Knittel K. Macpherson E. Boetius A. Brü Ckmann W. (2013a). Methane-Carbon Flow into the Benthic Food Web at Cold Seeps-A Case Study from the Costa Rica Subduction Zone. *PLoS One* 8:e74894. 10.1371/journal.pone.0074894 24116017 PMC3792092

[B67] Niemann H. Lösekann T. de Beer D. Elvert M. Nadalig T. Knittel K. (2006). Novel microbial communities of the Haakon Mosby mud volcano and their role as a methane sink. *Nature* 443 854–858. 10.1038/nature05227 17051217

[B68] Oksanen J. Blanchet F. G. Friendly M. Kindt R. Legendre P. Mcglinn D. (2019). *Vegan: Community Ecology Package. R Package Version 2.5-5. 2019.* Available at: https://CRAN.R-project.org/package=vegan [accessed December 9, 2019].

[B69] Orphan V. J. House C. H. Hinrichs K.-U. McKeegan K. D. DeLong E. F. (2002). Multiple archaeal groups mediate methane oxidation in anoxic cold seep sediments. *Proc. Natl. Acad. Sci. U. S. A.* 99 7663–7668. 10.1073/pnas.072210299 12032340 PMC124316

[B70] Panieri G. Bünz S. Fornari D. J. Escartin J. Serov P. Jansson P. (2017). An integrated view of the methane system in the pockmarks at Vestnesa Ridge, 79°N. *Mar. Geol.* 390 282–300. 10.1016/j.margeo.2017.06.006

[B71] Panieri G. Sen Gupta B. K. (2008). Benthic Foraminifera of the Blake Ridge hydrate mound, Western North Atlantic Ocean. *Mar. Micropaleontol.* 66 91–102. 10.1016/j.marmicro.2007.08.002

[B72] Portnov A. Vadakkepuliyambatta S. Mienert J. Hubbard A. (2016). Ice-sheet-driven methane storage and release in the Arctic. *Nat. Commun.* 7:10314. 10.1038/ncomms10314 26739497 PMC4729839

[B73] Quast C. Pruesse E. Yilmaz P. Gerken J. Schweer T. Yarza P. (2012). The SILVA ribosomal RNA gene database project: improved data processing and web-based tools. *Nucleic Acids Res.* 41 D590–D596. 10.1093/nar/gks1219 23193283 PMC3531112

[B74] Reeburgh W. S. (2007). Oceanic Methane Biogeochemistry. *Chem. Rev.* 107 486–513. 10.1021/CR050362V 17261072

[B75] Rey F. Rune Skjoldal H. (1987). *Consumption of silicic acid below the euphotic zone by sedimenting diatom blooms in the Barents Sea.* Available at: https://imr.brage.unit.no/imr-xmlui/bitstream/handle/11250/108035/m036p307.pdf?sequence=1 [Accessed July 11, 2019].

[B76] Rinke C. Schmitz-Esser S. Stoecker K. Nussbaumer A. D. Molnár D. A. Vanura K. (2006). &quot;Candidatus Thiobios zoothamnicoli,&quot; an ectosymbiotic bacterium covering the giant marine ciliate Zoothamnium niveum. *Appl. Environ. Microbiol.* 72 2014–2021. 10.1128/AEM.72.3.2014-2021.2006 16517650 PMC1393213

[B77] Roalkvam I. Dahle H. Chen Y. Jørgensen S. L. Haflidason H. Steen I. H. (2012). Fine-Scale Community Structure Analysis of ANME in Nyegga Sediments with High and Low Methane Flux. *Front. Microbiol.* 3:216. 10.3389/fmicb.2012.00216 22715336 PMC3375579

[B78] Roalkvam I. Jørgensen S. L. Chen Y. Stokke R. Dahle H. Hocking W. P. (2011). New insight into stratification of anaerobic methanotrophs in cold seep sediments. *FEMS Microbiol. Ecol.* 78 233–243. 10.1111/j.1574-6941.2011.01153.x 21676010

[B79] Robador A. Müller A. L. Sawicka J. E. Berry D. Hubert C. R. J. Loy A. (2016). Activity and community structures of sulfate-reducing microorganisms in polar, temperate and tropical marine sediments. *ISME J.* 10 796–809. 10.1038/ismej.2015.157 26359912 PMC4796921

[B80] Rossel P. E. Elvert M. Ramette A. Boetius A. Hinrichs K. U. (2011). Factors controlling the distribution of anaerobic methanotrophic communities in marine environments: Evidence from intact polar membrane lipids. *Geochim. Cosmochim. Acta* 75 164–184. 10.1016/j.gca.2010.09.031

[B81] Ruff S. E. Biddle J. F. Teske A. P. Knittel K. Boetius A. Ramette A. (2015). Global dispersion and local diversification of the methane seep microbiome. *Proc. Natl. Acad. Sci.* 112 4015–4020. 10.1073/pnas.1421865112 25775520 PMC4386351

[B82] Schoell M. (1988). Multiple origins of methane in the Earth. *Chem. Geol.* 71 1–10. 10.1016/0009-2541(88)9010190105

[B83] Schönfeld J. Alve E. Geslin E. Jorissen F. Korsun S. Spezzaferri S. (2012). The FOBIMO (FOraminiferal BIo-MOnitoring) initiative-Towards a standardised protocol for soft-bottom benthic foraminiferal monitoring studies. *Mar. Micropaleontol.* 9 1–13. 10.1016/j.marmicro.2012.06.001

[B84] Seeberg-Elverfeldt J. Schlüter M. Feseker T. Kölling M. (2005). Rhizon sampling of porewaters near the sediment-water interface of aquatic systems. *Limnol. Oceanogr. Methods* 3 361–371. 10.4319/lom.2005.3.361

[B85] Sen A. Aström E. K. L. Hong W. L. Portnov A. Waage M. Serov P. (2018). Geophysical and geochemical controls on the megafaunal community of a high Arctic cold seep. *Biogeosciences* 15 4533–4559. 10.5194/bg-15-45332018

[B86] Serov P. Vadakkepuliyambatta S. Mienert J. Patton H. Portnov A. Silyakova A. (2017). Postglacial response of Arctic Ocean gas hydrates to climatic amelioration. *Proc. Natl. Acad. Sci.* 114 6215–6220. 10.1073/pnas.1619288114 28584081 PMC5474808

[B87] Stahl D. A. de la Torre J. R. (2012). Physiology and Diversity of Ammonia-Oxidizing Archaea. *Annu. Rev. Microbiol* 66 83–101. 10.1146/annurev-micro-092611150128 22994489

[B88] Stokke R. Roalkvam I. Lanzen A. Haflidason H. Steen I. H. (2012). Integrated metagenomic and metaproteomic analyses of an ANME-1-dominated community in marine cold seep sediments. *Environ. Microbiol.* 14 1333–1346. 10.1111/j.1462-2920.2012.02716.x 22404914

[B89] Takishita K. Kakizoe N. Yoshida T. Maruyama T. (2010). Molecular Evidence that Phylogenetically Diverged Ciliates Are Active in Microbial Mats of Deep-Sea Cold-Seep Sediment. *J. Eukaryot. Microbiol.* 57 76–86. 10.1111/j.1550-7408.2009.00457.x 20002870

[B90] Takishita K. Tsuchiya M. Reimer J. D. Maruyama T. (2006). Molecular evidence demonstrating the basidiomycetous fungus Cryptococcus curvatus is the dominant microbial eukaryote in sediment at the Kuroshima Knoll methane seep. *Extremophiles* 10 165–169. 10.1007/s00792-005-0495497 16341819

[B91] Takishita K. Yubuki N. Kakizoe N. Inagaki Y. Maruyama T. (2007). Diversity of microbial eukaryotes in sediment at a deep-sea methane cold seep: surveys of ribosomal DNA libraries from raw sediment samples and two enrichment cultures. *Extremophiles* 11 563–576. 10.1007/s00792-007-0068-z 17426921

[B92] Timmers P. H. A. Widjaja-Greefkes H. C. A. Ramiro-Garcia J. Plugge C. M. Stams A. J. M. (2015). Growth and activity of ANME clades with different sulfate and sulfide concentrations in the presence of methane. *Front. Microbiol.* 6:988. 10.3389/fmicb.2015.00988 26441917 PMC4585129

[B93] Vaughn Barrie J. Cook S. Conway K. W. (2011). Cold seeps and benthic habitat on the Pacific margin of Canada. *Cont. Shelf Res.* 31 S85–S92. 10.1016/J.CSR.2010.02.013

[B94] Vigneron A. Alsop E. B. Cruaud P. Philibert G. King B. Baksmaty L. (2019). Contrasting Pathways for Anaerobic Methane Oxidation in Gulf of Mexico Cold Seep Sediments. *mSystems* 4:30834326 10.1128/msystems.0009118 PMC6392090 30834326

[B95] Vigneron A. Cruaud P. Pignet P. Caprais J.-C. Cambon-Bonavita M.-A. Godfroy A. (2013). Archaeal and anaerobic methane oxidizer communities in the Sonora Margin cold seeps, Guaymas Basin (Gulf of California). *ISME J.* 7 1595–1608. 10.1038/ismej.2013.18 23446836 PMC3721105

[B96] Vogt P. R. Crane K. Sundvor E. Max M. D. Pfirman S. L. (1994). Methane-generated(?) pockmarks on young, thickly sedimented oceanic crust in the Arctic: Vestnesa Ridge, Fram Strait. *Geology* 22 255–258. 10.1130/0091-7613(1994)022<0255:mgpoyt>2.3.co;2

[B97] Waage M. Portnov A. Serov P. Bünz S. Waghorn K. A. Vadakkepuliyambatta S. (2019). Geological Controls on Fluid Flow and Gas Hydrate Pingo Development on the Barents Sea Margin. *Geochemistry, Geophys. Geosystems* 20 630–650. 10.1029/2018GC007930

[B98] Webster G. Rinna J. Roussel E. G. Fry J. C. Weightman A. J. Parkes R. J. (2010). Prokaryotic functional diversity in different biogeochemical depth zones in tidal sediments of the Severn Estuary, UK, revealed by stable-isotope probing. *FEMS Microbiol. Ecol.* 72 179–197. 10.1111/j.1574-6941.2010.00848.x 20337706

[B99] Wegener G. Krukenberg V. Riedel D. Tegetmeyer H. E. Boetius A. (2015). Intercellular wiring enables electron transfer between methanotrophic archaea and bacteria. *Nature* 526 587–590. 10.1038/nature15733 26490622

[B100] Werne J. P. Baas M. Sinninghe Damsté J. S. (2002). Molecular isotopic tracing of carbon flow and trophic relationships in a methane-supported benthic microbial community. *Limnol. Oceanogr.* 47 1694–1701. 10.4319/lo.2002.47.6.1694

[B101] Wollenburg J. E. Mackensen A. (1998). Living benthic foraminifers from the central Arctic Ocean: Faunal composition, standing stock and diversity. *Mar. Micropaleontol.* 34 153–185. 10.1016/S0377-8398(98)000073

[B102] Yamamoto M. Takai K. (2011). Sulfur Metabolisms in Epsilon- and Gamma-Proteobacteria in Deep-Sea Hydrothermal Fields. *Front. Microbiol*. 2(192):192 10.3389/fmicb.2011.00192 21960986 PMC3176464

[B103] Yanagawa K. Sunamura M. Lever M. A. Morono Y. Hiruta A. Ishizaki O. (2011). Niche Separation of Methanotrophic Archaea (ANME-1 and-2) in Methane-Seep Sediments of the Eastern Japan Sea Offshore Joetsu. *Tetsuro Urabe Fumio Ina.* 28 118–129. 10.1080/01490451003709334

[B104] Yilmaz P. Parfrey L. W. Yarza P. Gerken J. Pruesse E. Quast C. (2014). The SILVA and “‘All-species Living Tree Project (LTP)”’ taxonomic frameworks. *Nucleic Acids Res.* 42 D643–D648. 10.1093/nar/gkt1209 24293649 PMC3965112

