# Supplementary material for: The Impact of Methane on Microbial Communities at Marine Arctic Gas Hydrate Bearing Sediment
Source: Front Microbiol. 2020 Sep 24;11:1932. doi: 10.3389/fmicb.2020.01932 (PMC7541813; doi:10.3389/fmicb.2020.01932)
Supplement: Supplementary file 1 [file Data_Sheet_1.docx]

Supplementary Material

# Supplementary Figures and Tables

## Supplementary Figures


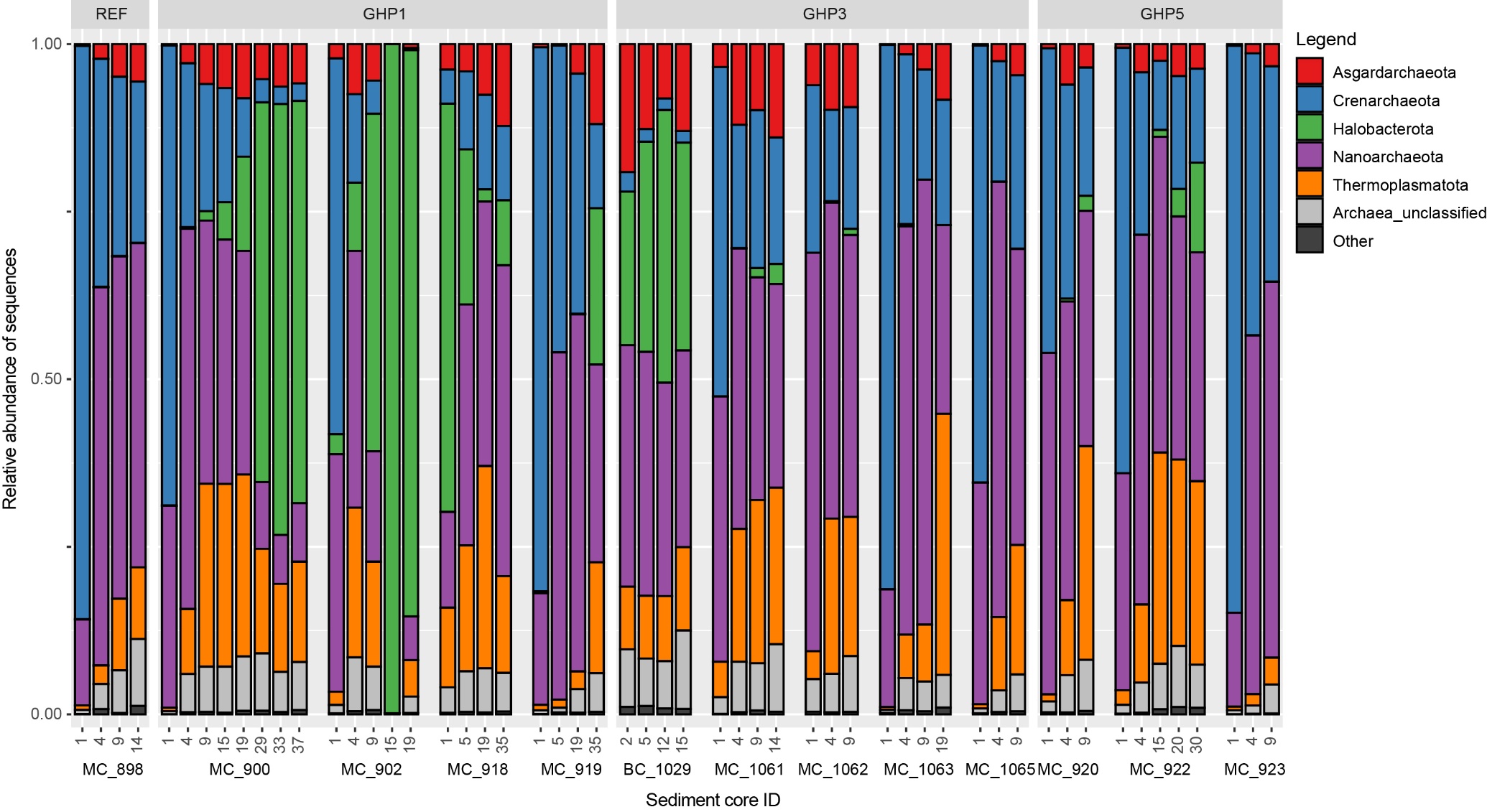


Figure SI 1: Relative abundances of sequences associated to archaeal taxa retrieved from sediments layers taken at the reference site (MC_898) and the GHPs 1, 3 and 5. A taxonomic group is abundant if it is associated in at least 1% of a sample’s sequences. “Unclassified” includes archaeal sequences that could not be assigned to a taxonomic group while “Other” refers to sequences associated to non-abundant taxonomic groups.


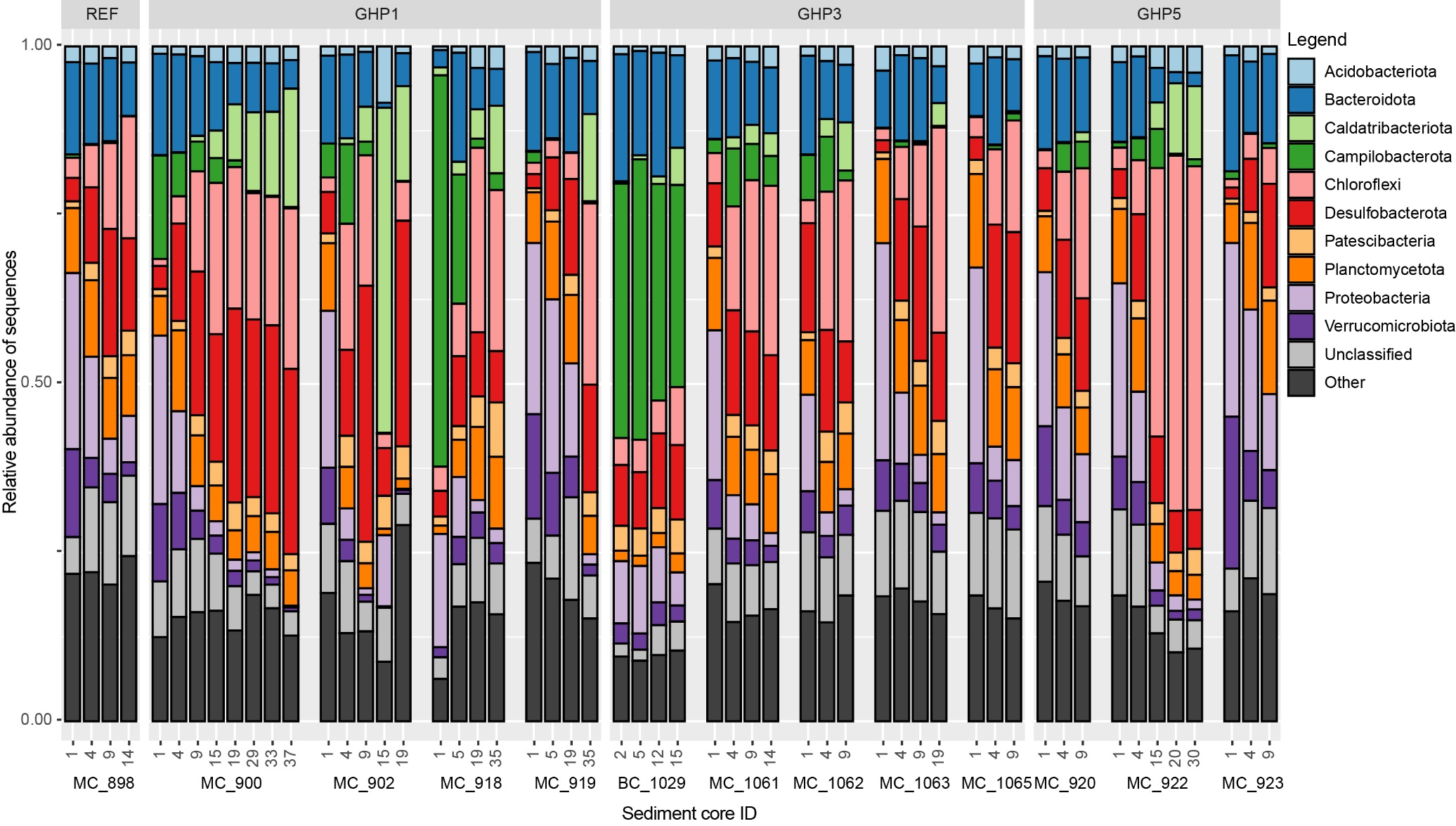


Figure SI 2: Relative abundances of sequences associated to bacterial taxa retrieved from sediments layers taken at the reference site (MC_898) and the GHPs 1, 3 and 5. A taxonomic group is abundant if it is associated in at least 1% of a sample’s sequences. “Unclassified” includes bacterial sequences that could not be assigned to a taxonomic group while “Other” refers to sequences associated to non-abundant taxonomic groups*.*


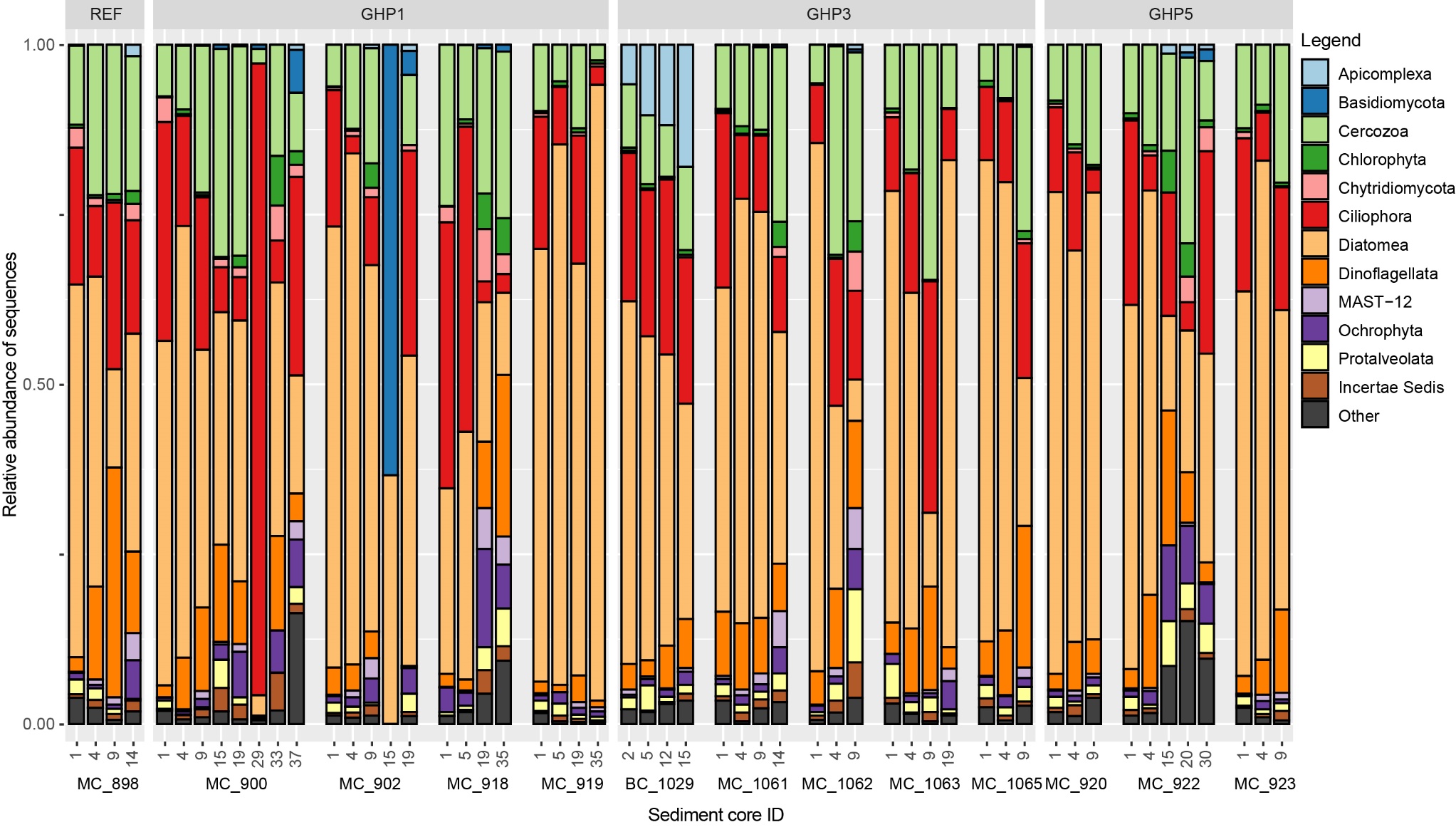


Figure SI 3: Relative abundances of sequences associated to eukaryotic taxa retrieved from sediments layers taken at the reference site (MC_898) and the GHPs 1, 3 and 5. A taxonomic group is abundant if it is associated in at least 1% of a sample’s sequences. “Unclassified” includes eukaryotic sequences that could not be assigned to a taxonomic group while “Other” refers to sequences associated to non-abundant taxonomic groups.


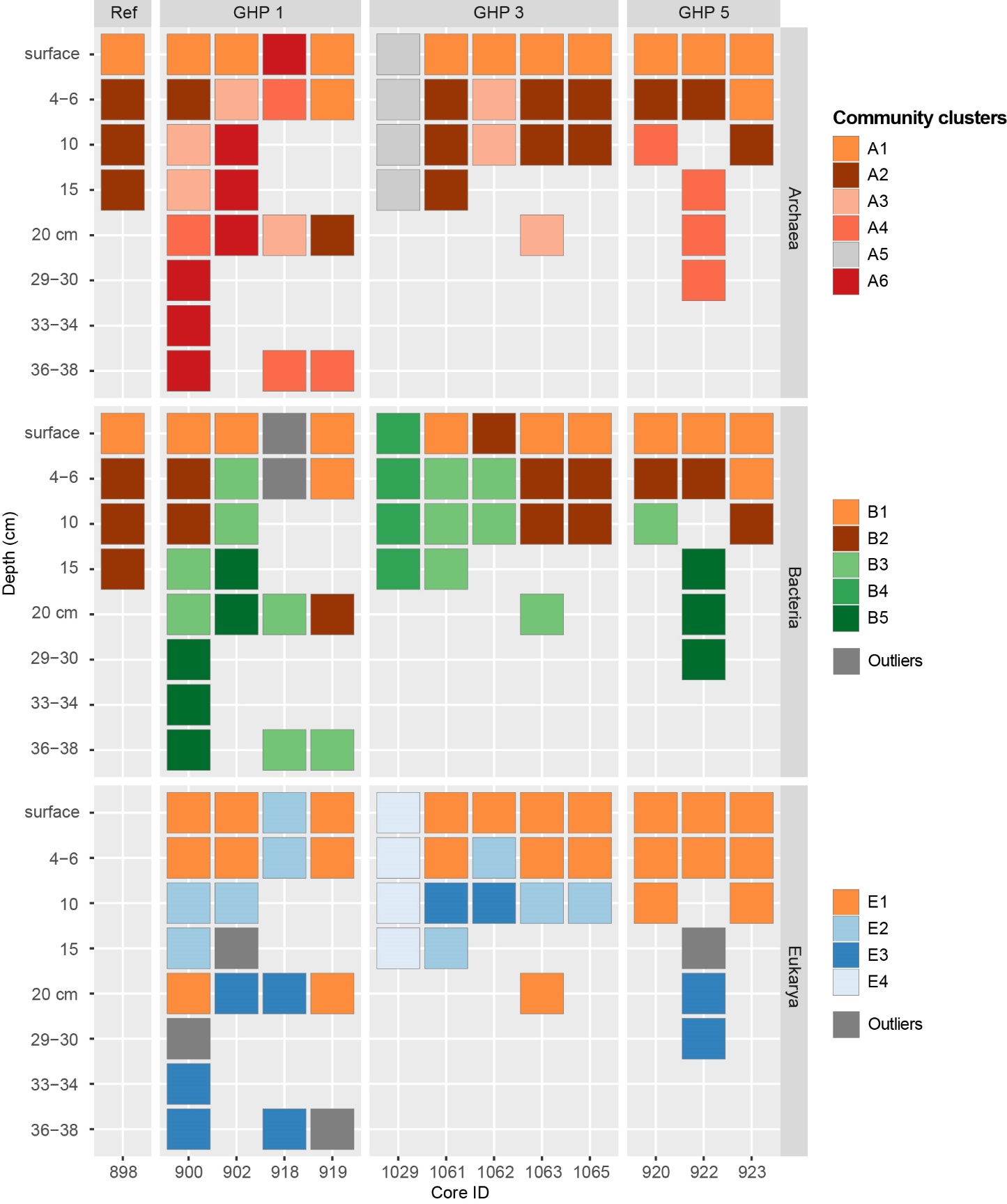


Figure SI 4: Spatial distribution of the archaeal, bacterial and eukaryotic clusters identified in Figures 4, 6 and 7 at the different sediment layers of the reference site and GHP 1, GHP 2 and GHP 3.


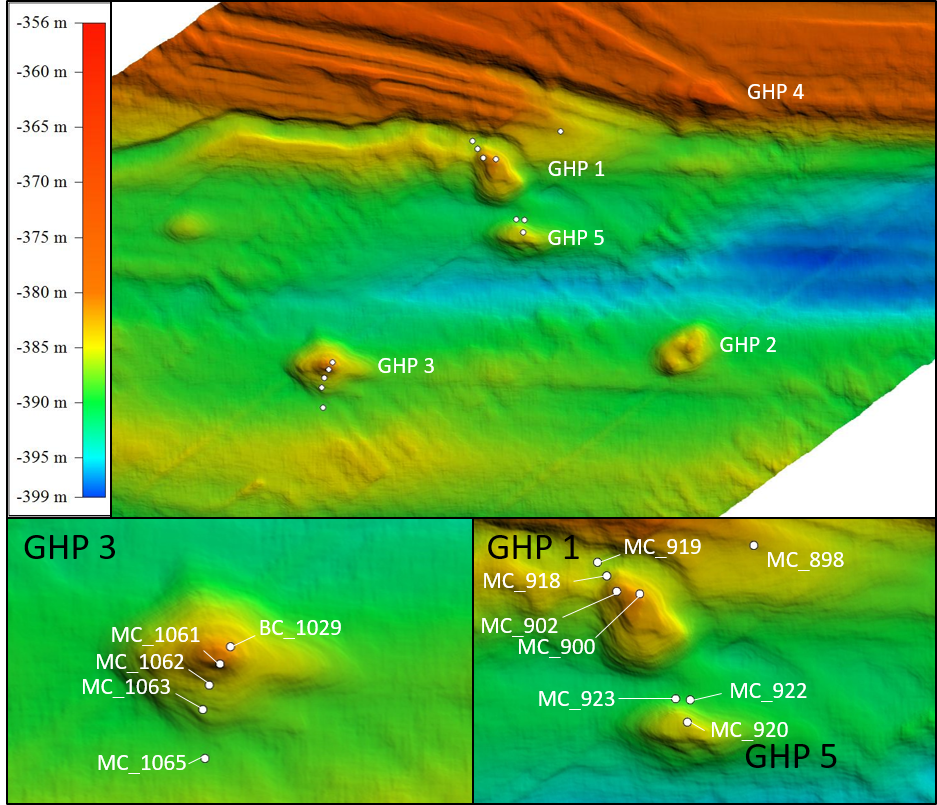


Figure SI 5: Figure SI 5 Linear Relationship (R2= 0.935) between the logarithmic (base 10) number of Sulfurimonas spp. reads (resampled) and the density of agglutinated foraminifera cells in GHP 1 surface sample. The Sulfurimonas spp. reads are derived from sequencing results whereas the number of foraminifera cells were counted under a stereoscopic microscope.

## Supplementary Tables

Table SI 1 The table presents sampling depths within each core for GHP 1. Values for methane (CH_4_), sulfate (SO_4_), sulfide (HS^-^) and porosity at these sediment depths are given. Some depths were not measured and are indicated with -. The presented depths correspond to the sediment layers from which the DNA was extracted and sequenced.

| Core ID | MC_900 | | | | | | | | MC_902 | | | | | MC_918 | | | | MC_919 | | | |
| --- | --- | --- | --- | --- | --- | --- | --- | --- | --- | --- | --- | --- | --- | --- | --- | --- | --- | --- | --- | --- | --- |
| Depth (cmbsf) | 1 | 5 | 9 | 15 | 19 | 29 | 33 | 37 | 1 | 5 | 9 | 15 | 19 | 1 | 5 | 19 | 35 | 1 | 5 | 19 | 35 |
| CH4 (µM) | 4.73 | 13.6 | 20.7 | 32.7 | 59.3 | 107 | 132 | 170 | 6.73 | 13.2 | 41.8 | 152 | 1110 | 1.86 | 14.3 | 23.9 | 81 | 0.61 | 2.13 | 6.23 | 12.2 |
| SO4 (mM) | 28.4 | 27.1 | 24.9 | 24.4 | 21.4 | 13.7 | 2.72 | - | 20.2 | 12.5 | 15.7 | 4.13 | 0.93 | 27.8 | 26.6 | 25.9 | 22.3 | 28 | 27.5 | 27.4 | 26.1 |
| HS- (µM) | 0 | 0 | 559 | 1131 | 1719 | 1928 | 4559 | - | 849 | 1923 | 2079 | 2045 | 1986 | 16.5 | 82.9 | 248 | 2193 | 0 | 0 | 0 | 0 |
| Porosity | 0.86 | 0.76 | 0.74 | 0.78 | 0.67 | 0.73 | 0.65 | 0.65 | 0.85 | 0.64 | 0.64 | 0.56 | 0.7 | 0.83 | 0.71 | 0.73 | 0.67 | 0.88 | 0.83 | 0.78 | 0.69 |

Table SI 2 The table presents sampling depths within each core for GHP 3. Values for methane (CH_4_), sulfate (SO_4_), sulfide (HS^-^) and porosity at these sediment depths are given. Some depths were not measured and are indicated with -. The presented depths correspond to the sediment layers from which the DNA was extracted and sequenced.

| Core ID | MC_1061 | | | | MC_1062 | | | MC_1065 | | |
| --- | --- | --- | --- | --- | --- | --- | --- | --- | --- | --- |
| Depth (cmbsf) | 1 | 4 | 9 | 14 | 1 | 4 | 9 | 1 | 4 | 9 |
| CH4 (µM) | 1.91 | 9.03 | 9.73 | 12.6 | 1.91 | 8.16 | 14.4 | 7.68 | 7.22 | 6.75 |
| SO4 (mM) | 27.7 | 27.3 | 25.7 | 23.8 | 27.7 | 27.2 | 26.5 | 27.3 | 27.3 | 27.3 |
| HS- (µM) | - | - | - | - | - | - | - | - | - | - |
| Porosity | 0.74 | 0.69 | 0.56 | 0.36 | 0.72 | 0.61 | 0.64 | 0.36 | 0.43 | 0.51 |

Table SI 3 The table presents sampling depths within each core for the reference site (MC_898) and the GHP 5. Values for methane (CH_4_), sulfate (SO_4_), sulfide (HS^-^) and porosity at these sediment depths are given. Some depths were not measured and are indicated with -. The presented depths correspond to the sediment layers from which the DNA was extracted and sequenced.

| Core ID | MC_898 | | | | MC_920 | | | MC_922 | | | | | MC_923 | | |
| --- | --- | --- | --- | --- | --- | --- | --- | --- | --- | --- | --- | --- | --- | --- | --- |
| Depth (cmbsf) | 1 | 4 | 9 | 14 | 1 | 4 | 9 | 1 | 4 | 15 | 20 | 30 | 1 | 4 | 9 |
| CH4 (µM) | 0.69 | 1.13 | 0.98 | 2.32 | 1.28 | 8.89 | 18.4 | 0.78 | 0 | 8.69 | 18.3 | 30 | 0.37 | 1.2 | 1.95 |
| SO4 (mM) | 28.5 | 27.4 | 26.7 | - | - | - | - | - | - | - | - | - | - | - | - |
| HS- (µM) | 0 | 0 | 0 | 0 | 0 | 0 | 0 | 0 | 0 | 0 | 29.2 | 3077 | 0 | 0 | 0 |
| Porosity | 0.87 | 0.72 | 0.6 | 0.56 | 0.83 | 0.72 | 0.63 | 0.75 | 0.51 | 0.58 | 0.52 | 0.6 | 0.95 | 0.75 | 0.81 |

Table SI 4: This table shows the depths for chemical analyses and visual observation of the sites. Are presented in the table a list of sediments layers, from which DNA was extracted and sequenced, collected from different cores at the reference site and at the GHP 1. Are also set forth in the table measured concentrations of iron (Fe), and dissolved inorganic carbon (DIC), alkalinity and a general description of the seafloor where the sediment cores were taken.

| Location | Core ID | Depth | Fe (µM) | alkalinity | DIC (mM) | Site Description |
| --- | --- | --- | --- | --- | --- | --- |
|  |  | (cmbsf) |  | (mM) |  |  |
| RS | MC_898 | 1 | 0.09 | 0.66 | 2.35 | Seafloor is muddy and evidences of bioturbation. Gas flares were absent and were visible some siboglinids. |
|  |  | 4 | 132.68 | 1.35 | 2.65 |  |
|  |  | 9 | 74.04 | 1.47 | 2.65 |  |
|  |  | 14 | - | - | - |  |
| GHP 1 | MC_900 | 1 | 0 | 2.55 | 5.62 | The core was taken above a dense field of siboglinids. Bacterial mats and carbonate crusts colonized by anemones and sponges are visible in the vicinity. |
|  |  | 5 | 26.12 | 3.65 | 3.96 |  |
|  |  | 9 | 0.26 | 7.02 | 5.9 |  |
|  |  | 15 | 0.09 | - | 4.81 |  |
|  |  | 19 | 0 | 13.40 | 7.37 |  |
|  |  | 29 | 0 | - | 11.92 |  |
|  |  | 33 | 2.10 | 30.65 | 17.3 |  |
|  |  | 37 | - | - | - |  |
|  | MC_902 | 1 | 1.88 | - | 11.93 | Similarly composed to core 900, the core 902 was taken in an area with a higher density of bacterial mats than core 900. |
|  |  | 5 | 0.52 | 25.31 | 18.08 |  |
|  |  | 9 | 0.09 | 36.00 | 15.42 |  |
|  |  | 15 | 0.81 | - | 24.15 |  |
|  |  | 19 | 0 | 43.67 | 27.12 |  |
|  | MC_918 | 1 | 1.67 | - | 2.52 | Bacterial mats and patches of siboglinids remain visible. |
|  |  | 5 | 0 | 4.23 | 3.39 |  |
|  |  | 19 | 0 | - | 4.85 |  |
|  |  | 35 | 0 | 1.57 | 6.75 |  |
|  | MC_919 | 1 | 0 | - | 2.77 | Bacterial mats, sponges and corals, and siboglinids remain visible. |
|  |  | 5 | 143.38 | - | 3.09 |  |
|  |  | 19 | 210.14 | - | 3.3 |  |
|  |  | 35 | - | - | - |  |

Table SI 5: This table shows the depths for chemical analyses and visual observation of the sites. Are presented in the table a list of sediments layers, from which DNA was extracted and sequenced, collected from different cores at the GHP 3 and 5. Are also set forth in the table measured concentrations of iron (Fe), alkalinity and a general description of the seafloor where the sediment cores were taken. Geochemical data for BC_1029 and MC_1063 were collected from Hong et al., 2020.

| Location | Core ID | Depth | Fe (µM) | alkalinity | Site Description |
| --- | --- | --- | --- | --- | --- |
|  |  | (cmbsf) |  | (mM) |  |
| GHP 3 | BC_1029 | 2.5 | 1.93 | 2.62 | This blade core was taken using a ROV above a gas emitting flare. |
|  |  | 5 | 0.43 | 3.10 |  |
|  |  | 12 | 0 | 21.17 |  |
|  |  | 15 | 0 | 25.79 |  |
|  | MC_1061 | 1 | - | - | - |
|  |  | 4 | - | 7.80 |  |
|  |  | 9 | - | - |  |
|  |  | 14 | - | - |  |
|  | MC_1063 | 1 | 112.71 | - | - |
|  |  | 4 | 83.93 | - |  |
|  |  | 9 | 18.09 | - |  |
|  |  | 19 | 0.64 | - |  |
| GHP 5 | MC_920 | 1 | - | - | Anemones and sponges on hard surfaces |
|  |  | 4 | - | - |  |
|  |  | 9 | - | - |  |
|  | MC_922 | 1 | 3.98 | 4.59 | Same biology as core 920 and fishes are visible at core 922 |
|  |  | 4 | 9.97 | - |  |
|  |  | 15 | - | - |  |
|  |  | 20 | 0.13 | - |  |
|  |  | 30 | 0 | 2.79 |  |
|  | MC_923 | 1 | 24.19 | - | Hard surfaces becomes more rare |
|  |  | 4 | 123.69 | 11.62 |  |
|  |  | 9 | - | - |  |

Table SI 6: Primers sets used to target variable regions of 16S rDNA of prokaryotes and 18S rDNA of eukaryotes in the GHP sediment samples and their PCR product length in base pairs (bp).

| Primer Name | Primer sequence | Amplicon size | Reference |
| --- | --- | --- | --- |
| *Bacteria, V3-V4 16S rRNA* | | | |
| Bakt_341F | CCTACGGGNGGCWGCAG | 464 bp | (Klindworth et al., 2013) |
| Bakt_805R | GACTACHVGGGTATCTAATCC |  | (Klindworth et al., 2013) |
| *Archaea, V3-V4 16S rRNA* | | | |
| A519F | CAGCMGCCGCGGTAA | 387 bp | (Alm et al., 1996; Jorgensen et al., 2012) |
| A906 | CAATTCMTTTAAGTTTC |  | (Alm et al., 1996) |
| *Eukarya, V4 18S rRNA* | | | |
| 18S-574f | CGGTAAYTCCAGCTCYV | 323 bp | (Hugerth et al., 2014) |
| 18S-897r | TCYDAGAATTYCACCTCT |  | (Hugerth et al., 2014) |

Table SI 7: The table presents the original numbers of paired reads before and after quality filtering, in addition to the total numbers of OTUs formed, of OTUs assigned to their domain and of OTUs retained after rarefactions steps. Prokaryotic libraries were rarefied at 8700 and 4700 sequences for the Archaea and Bacteria, respectively. Eukaryotic libraries were rarefied at 1300 sequences.

| Domain | Paired reads prior to QC (n) | Paired reads after QC (n) | OTUs formed (n) | OTUs assigned to their domain (n) | OTUs after rarefactions (n) |
| --- | --- | --- | --- | --- | --- |
| Archaea | 3 195 913 | 2 218 007 | 10 103 | 8129 | 7131 |
| Bacteria | 2 506 231 | 1 892 253 | 39 387 | 36301 | 26761 |
| Eukarya | 3 491 383 | 2 778 342 | 10 232 | 8184 | 1376 |

Table SI 8: Alpha diversity of the OTUs at different sediment layers selected from cores taken at the reference site and at gas hydrate pingo 1 (GHP). Shannon’s (H) and Inverse Simpson’s (D) diversity metrics were used. Prior to the analyses, OTUs that were not assigned to their respective domain or associated to Metazoa were removed from the analyses.

| Location | Core ID | Depth | Archaea | | Bacteria | | Eukarya | |
| --- | --- | --- | --- | --- | --- | --- | --- | --- |
|  |  | (cmbsf) | H | D | H | D | H | D |
|  |  |  |  |  |  |  |  |  |
| Reference Site | MC_898 | 1 | 3.90 | 0.76 | 9.63 | 1.00 | 6.11 | 0.94 |
|  |  | 4 | 7.55 | 0.96 | 9.70 | 1.00 | 4.88 | 0.79 |
|  |  | 9 | 7.36 | 0.96 | 9.71 | 1.00 | 4.62 | 0.88 |
|  |  | 14 | 7.67 | 0.98 | 9.30 | 1.00 | 6.83 | 0.98 |
|  |  |  |  |  |  |  |  |  |
| GHP 1 | MC_900 | 1 | 4.31 | 0.80 | 9.07 | 0.99 | 6.74 | 0.96 |
|  |  | 5 | 7.35 | 0.97 | 9.35 | 0.99 | 4.14 | 0.85 |
|  |  | 9 | 7.20 | 0.97 | 9.59 | 0.99 | 2.31 | 0.44 |
|  |  | 15 | 7.38 | 0.97 | 9.80 | 1.00 | 0.42 | 0.06 |
|  |  | 19 | 7.16 | 0.98 | 8.37 | 0.98 | 6.39 | 0.96 |
|  |  | 29 | 4.37 | 0.80 | 7.41 | 0.97 | 2.23 | 0.51 |
|  |  | 33 | 3.79 | 0.73 | 7.23 | 0.97 | 3.59 | 0.82 |
|  |  | 37 | 4.03 | 0.76 | 6.27 | 0.93 | 4.67 | 0.85 |
|  | MC_902 | 1 | 5.83 | 0.86 | 9.98 | 1.00 | 5.76 | 0.94 |
|  |  | 5 | 6.99 | 0.97 | 8.83 | 0.99 | 3.51 | 0.75 |
|  |  | 9 | 4.70 | 0.79 | 6.39 | 0.90 | 2.06 | 0.50 |
|  |  | 15 | 0.17 | 0.03 | 3.87 | 0.76 | 2.54 | 0.75 |
|  |  | 19 | 1.73 | 0.37 | 5.39 | 0.92 | 4.88 | 0.94 |
|  | MC_918 | 1 | 3.96 | 0.65 | 5.79 | 0.90 | 5.76 | 0.94 |
|  |  | 5 | 6.93 | 0.94 | 8.93 | 0.99 | 5.62 | 0.93 |
|  |  | 19 | 7.34 | 0.97 | 9.21 | 0.99 | 7.09 | 0.98 |
|  |  | 35 | 6.64 | 0.97 | 8.06 | 0.98 | 6.95 | 0.97 |
|  | MC_919 | 1 | 4.12 | 0.75 | 9.01 | 0.99 | 6.10 | 0.94 |
|  |  | 5 | 5.84 | 0.91 | 9.54 | 1.00 | 5.39 | 0.90 |
|  |  | 19 | 6.45 | 0.94 | 9.77 | 0.99 | 2.98 | 0.55 |
|  |  | 35 | 6.05 | 0.94 | 8.09 | 0.97 | 2.68 | 0.61 |
|  |  |  |  |  |  |  |  |  |

Table SI 9: Alpha diversity of the OTUs at different sediment layers selected from cores taken at the gas hydrate pingos 3 and 5 (GHPs 3 and 5). Shannon’s (H) and Inverse Simpson’s (D) and diversity metrics were used. Prior to the analyses, OTUs that were not assigned to their respective domain or associated to Metazoa were removed from the analyses.

| Location | Core ID | Depth | Archaea | | Bacteria | | Eukarya | |
| --- | --- | --- | --- | --- | --- | --- | --- | --- |
|  |  | (cmbsf) | H | D | H | D | H | D |
|  |  |  |  |  |  |  |  |  |
| GHP 3 | BC_1029 | 2.5 | 6.93 | 0.97 | 7.01 | 0.95 | 6.00 | 0.94 |
|  |  | 5 | 6.79 | 0.96 | 6.90 | 0.94 | 6.00 | 0.95 |
|  |  | 12 | 6.20 | 0.92 | 7.59 | 0.96 | 6.19 | 0.95 |
|  |  | 15 | 6.04 | 0.95 | 6.79 | 0.94 | 2.21 | 0.39 |
|  | MC_1061 | 1 | 6.29 | 0.92 | 10.36 | 1.00 | 5.58 | 0.90 |
|  |  | 4 | 6.83 | 0.96 | 9.21 | 0.99 | 4.09 | 0.82 |
|  |  | 9 | 6.95 | 0.96 | 8.82 | 0.99 | 4.14 | 0.77 |
|  |  | 14 | 6.72 | 0.96 | 8.98 | 0.99 | 2.27 | 0.41 |
|  | MC_1062 | 1 | 7.49 | 0.97 | 9.44 | 0.99 | 4.64 | 0.89 |
|  |  | 4 | 7.39 | 0.97 | 9.31 | 0.99 | 4.48 | 0.80 |
|  |  | 9 | 7.22 | 0.97 | 8.91 | 0.99 | 7.49 | 0.99 |
|  | MC_1063 | 1 | 3.97 | 0.76 | 9.89 | 1.00 | 5.74 | 0.90 |
|  |  | 4 | 8.20 | 0.97 | 9.88 | 1.00 | 3.95 | 0.70 |
|  |  | 9 | 7.43 | 0.97 | 9.93 | 0.99 | 6.16 | 0.96 |
|  |  | 19 | 6.48 | 0.96 | 9.19 | 0.99 | 5.67 | 0.91 |
|  | MC_1065 | 1 | 5.08 | 0.84 | 10.01 | 1.00 | 5.96 | 0.94 |
|  |  | 4 | 7.41 | 0.96 | 9.83 | 1.00 | 4.44 | 0.87 |
|  |  | 9 | 7.21 | 0.97 | 9.75 | 1.00 | 2.84 | 0.58 |
|  |  |  |  |  |  |  |  |  |
| GHP 5 | MC_920 | 1 | 6.39 | 0.91 | 9.63 | 1.00 | 6.10 | 0.92 |
|  |  | 4 | 7.58 | 0.97 | 9.53 | 1.00 | 4.60 | 0.85 |
|  |  | 9 | 7.50 | 0.98 | 9.88 | 1.00 | 5.85 | 0.92 |
|  | MC_922 | 1 | 5.61 | 0.85 | 10.56 | 1.00 | 5.64 | 0.93 |
|  |  | 4 | 7.56 | 0.97 | 9.91 | 1.00 | 2.34 | 0.43 |
|  |  | 15 | 7.35 | 0.98 | 8.90 | 0.99 | 2.69 | 0.62 |
|  |  | 20 | 7.66 | 0.98 | 7.24 | 0.96 | 5.28 | 0.84 |
|  |  | 30 | 7.23 | 0.97 | 6.98 | 0.96 | 6.52 | 0.96 |
|  | MC_923 | 1 | 3.68 | 0.73 | 9.38 | 0.99 | 6.03 | 0.94 |
|  |  | 4 | 6.66 | 0.93 | 9.85 | 1.00 | 4.22 | 0.84 |
|  |  | 9 | 7.15 | 0.96 | 9.38 | 0.99 | 5.49 | 0.91 |
|  |  |  |  |  |  |  |  |  |

Table SI 10: Number of living foraminifera cells identified in the first centimeters (0-2 cmbsf) of the reference core (MC_898) and GHP1 cores. Names with an asterisk corresponds to agglutinated species.

|  | *Bathysiphon* sp.* | *Buccella frigida* | *Cassidulina neoteretis* | *Cibicides lobatulus* | *Recurvoides turbinatum** | *Astrononion gallawayi* | *Fissurina* sp. | *Globobulimina auriculata* | *Haplophragmoides subglobusus** | *Islandiella norcrossi* | *Melonis barleeanum* | *Nonionella labradorica* | *Pullenia bulloides* | *Quinqueloculina* *seminula* | *Reophax guttifera** | *Reophax scorpiurus** | *Stainforthia loeblichi* | *Trifarina angulosa* | *Trifarina earlandi* | *Uvigerina peregrina* | TOTAL |
| --- | --- | --- | --- | --- | --- | --- | --- | --- | --- | --- | --- | --- | --- | --- | --- | --- | --- | --- | --- | --- | --- |
| MC_898 | 2 |  | 4 | 12 | 2 |  |  |  |  |  | 13 |  | 2 | 3 |  | 21 |  |  | 1 |  | 58 |
| MC_900 | 1 | 2 | 2 |  | 1 |  |  |  |  |  | 8 |  |  |  |  | 1 |  |  |  |  | 14 |
| MC_902 |  |  | 4 | 4 | 3 | 3 | 7 |  |  | 3 | 5 | 10 |  |  |  | 9 |  |  |  | 1 | 49 |
| MC_918 |  | 6 |  | 1 |  | 4 |  |  |  | 2 | 5 | 2 | 2 |  | 1 |  | 1 |  |  |  | 24 |
| MC_919 |  | 7 | 1 | 5 | 1 | 18 |  | 3 | 6 | 2 | 9 | 10 |  |  |  | 12 |  | 1 |  |  | 75 |

# Bibliography

Alm, E. W., Oerther, D. B., Larsen, N., Stahl, D. A., and Raskin, L. (1996). The oligonucleotide probe database. *Appl. Environ. Microbiol.* 62, 3557–9. Available at: http://www.ncbi.nlm.nih.gov/pubmed/8837410 [Accessed December 5, 2018].

Hong, W. L., Latour, P., Sauer, S., Sen, A., Gilhooly, W. P., Lepland, A., et al. (2020). Iron cycling in Arctic methane seeps. *Geo-Marine Lett.*, 1–11. doi:10.1007/s00367-020-00649-5.

Hugerth, L. W., Muller, E. E. L., Hu, Y. O. O., Lebrun, L. A. M., Roume, H., Lundin, D., et al. (2014). Systematic Design of 18S rRNA Gene Primers for Determining Eukaryotic Diversity in Microbial Consortia. *PLoS One* 9, e95567. doi:10.1371/journal.pone.0095567.

Jorgensen, S. L., Hannisdal, B., Lanzén, A., Baumberger, T., Flesland, K., Fonseca, R., et al. (2012). Correlating microbial community profiles with geochemical data in highly stratified sediments from the Arctic Mid-Ocean Ridge. *Proc. Natl. Acad. Sci. U. S. A.* 109, E2846-55. doi:10.1073/pnas.1207574109.

Klindworth, A., Pruesse, E., Schweer, T., Peplies, J., Quast, C., Horn, M., et al. (2013). Evaluation of general 16S ribosomal RNA gene PCR primers for classical and next-generation sequencing-based diversity studies. *Nucleic Acids Res.* 41, e1–e1. doi:10.1093/nar/gks808.
